# Assays to Detect β-Tubulin Codon 200 Polymorphism in *Trichuris trichiura* and *Ascaris lumbricoides*

**DOI:** 10.1371/journal.pntd.0000397

**Published:** 2009-03-24

**Authors:** Aissatou Diawara, Lesley J. Drake, Richard R. Suswillo, Jimmy Kihara, Donald A. P. Bundy, Marilyn E. Scott, Carli Halpenny, J. Russell Stothard, Roger K. Prichard

**Affiliations:** 1 Institute of Parasitology, McGill University, Ste. Anne de Bellevue, Quebec, Canada; 2 The Partnership for Child Development, Imperial College School of Medicine, London, United Kingdom; 3 Kenya Medical Research Institute, Nairobi, Kenya; 4 World Bank, Washington D.C., United States of America; 5 Natural History Museum, London, United Kingdom; Queensland Institute for Medical Research, Australia

## Abstract

**Background:**

The soil-transmitted helminths (STH) *Ascaris lumbricoides* and *Trichuris trichiura* are gastrointestinal parasites causing many disabilities to humans, particularly children. The benzimidazole (BZ) drugs, albendazole (ALB) and mebendazole (MBZ), are commonly used for mass treatment for STH. Unfortunately, there is concern that increased use of anthelmintics could select for resistant populations of these human parasites. In veterinary parasites, and lately in filarial nematodes, a single amino acid substitution from phenylalanine to tyrosine, known to be associated with benzimidazole resistance, has been found in parasite β-tubulin at position 200. We have developed pyrosequencer assays for codon 200 (TTC or TAC) in *A. lumbricoides* and *T. trichiura* to screen for this single nucleotide polymorphism (SNP).

**Method and Findings:**

Pyrosequencing assays were developed and evaluated for detecting the TTC or TAC SNP at codon 200 in β-tubulin in *A. lumbricoides* and *T. trichiura*. Genomic DNA from individual worms, eggs isolated from individual adult worms or from fecal samples with known treatment history and origin, were sequenced at β-tubulin by pyrosequencing, and genotypes were confirmed by conventional sequencing. The assays were applied to adult worms from a benzimidazole-naïve population in Kenya. Following this, these assays were applied to individual worms and pooled eggs from people in East Africa (Uganda and Zanzibar) and Central America (Panama) where mass anthelmintic drug programs had been implemented. All *A. lumbricoides* samples were TTC. However, we found 0.4% homozygous TAC/TAC in *T. trichiura* worms from non-treated people in Kenya, and 63% of *T. trichiura* egg pools from treated people in Panama contained only TAC.

**Conclusion:**

Although the codon 200 TAC SNP was not found in any of the *A. lumbricoides* samples analyzed, a rapid genotyping assay has been developed that can be used to examine larger populations of this parasite and to monitor for possible benzimidazole resistance development. The TAC SNP at codon 200, associated with benzimidazole resistance in other nematodes, does occur in *T. trichiura*, and a rapid assay has been developed to allow populations of this parasite to be monitored for the frequency of this SNP. Sample sizes were small, anthelmintic efficacy was not assessed, and treated and non-treated samples were from different locations, so these frequencies cannot be extrapolated to other populations of *T. trichiura* or to a conclusion about resistance to treatment. The occurrence of the TAC SNP at codon 200 of β-tubulin in *T. trichiura* may explain why benzimidazole anthelmintics are not always highly effective against this species of STH. These assays will be useful in assessing appropriate treatment in areas of high *T. trichiura* prevalence and in monitoring for possible resistance development in these STH.

## Author Summary

The soil-transmitted helminths *Ascaris lumbricoides* and *Trichuris trichiura* are gastrointestinal nematodes causing many disabilities in tropical parts of the developing world. Control programs, such as “The Focussing Resources on Effective School Health” (FRESH) Partnership, have been implemented to remove human soil-transmitted nematodes through large-scale use of benzimidazole anthelmintic drugs for school-aged children in developing countries. The benzimidazole drugs albendazole and mebendazole are commonly used as a single annual treatment in areas where the burden is high. In veterinary nematodes, repeated use of these anthelmintics has selected for resistant populations. Resistance to benzimidazoles is commonly associated with a single amino acid substitution from phenylalanine to tyrosine in the β-tubulin gene at position 200. In this study, we have developed pyrosequencing assays for codon 200 in *A. lumbricoides* and *T. trichiura* to screen for this single nucleotide polymorphism (SNP) in β-tubulin. The 200Tyr SNP was detected at low frequency in *T. trichiura* from non-treated people from Kenya and at high frequency in *T. trichiura* from treated people from Panama. The presence of the resistance-associated SNP may play a role in the sometimes low and variable efficacy of benzimidazole anthelmintics against *T. trichiura*.

## Introduction

The soil-transmitted helminths (STH) are gastrointestinal nematodes widely distributed throughout the tropical and subtropical parts of the developing world. More than a billion people are infected with at least one species and 300 million are estimated to have severe infections with more than one of these parasites [1]. Infection often causes chronic disability but in certain instances may precipitate death. School age children are the most at risk of infection with STH and can show symptoms of malnourishment, experience growth stunting and intellectual retardation, with cognitive and educational deficits [1]. Control programs such as the “Focussing Resources on Effective School Health” partnership (FRESH) have been implemented in endemic countries to reduce the morbidity of school-aged children by using a single annual treatment with benzimidazole drugs : either albendazole or mebendazole [2].

BZ drugs are broad spectrum anthelmintics that bind to β-tubulin, causing interference with tubulin polymerization and destabilization of microtubules [3]. Mass drug administration (MDA) programs reduce the incidence and intensity of infections; however they can also cause a selection pressure on the parasites to develop resistance. Many studies have demonstrated that the widespread and frequent use of anthelmintic drugs in veterinary nematodes has led to the development of resistance [4],[5]. This resistance is usually due to a single nucleotide polymorphism (SNP) which causes an amino acid substitution from phenylalanine (Phe, TTC) to tyrosine (Tyr, TAC) in parasite β-tubulin at codon 200 [6]. A similar SNP at codon 167 (Phe167Tyr) or a glutamate to alanine change at codon 198 (Glu198Ala) can also occasionally be associated with benzimidazole resistance [7]. In human STH, and especially in hookworms, reports have suggested the development of benzimidazole resistance but failed to provide conclusive evidence [8]. If resistance against albendazole and mebendazole occurs, it will be a major threat to the mass de-worming programs in developing countries.

The objectives of this research were: (i) to determine the genomic sequences of the β-tubulin around codon 200 in *A. lumbricoides* and *T. trichiura*, (ii) to develop pyrosequencing assays for the detection of phenylalanine 200 or tyrosine 200 in the β-tubulin gene of each of these nematodes, and (iii) to assay DNA of individual adult worms and pooled eggs from the field to determine whether the Phe200Tyr SNP can be found and to obtain initial assessments of the frequency in areas that are either naïve to benzimidazole treatment or have been subject to benzimidazole treatment.

## Materials and Methods

### Parasite Material

In this study, all parasite samples were from school-age children who have been infected naturally by *A. lumbricoides* and/or *T. trichiura*. Ethical approval was obtained from the Research Ethics Board, McGill University, the London School of Hygiene and Tropical Medicine and the Kenya Medical Research Institute. Samples of adult worms and/or eggs were collected from different locations with different treatment histories. Individual adult worms, 39 *T. trichiura* (20 males and 19 females) and 38 *A. lumbricoides* (19 males and 19 females), were collected from children from Kisumu, Kenya (latitude: 00°03′ S, longitude: 35°5′ E). These children were naïve for anthelmintic treatment; however, they received a single dose of “Combantrin” (pyrantel) in order to expel the adult worms in feces. These worms were considered as control parasites and stored at −80°C until needed.

Faecal samples with *A. lumbricoides* and *T. trichiura* eggs were collected in Panama in the Comarca Ngobe Bugle region (latitude: 8°3′ N, longitude: 86°12′ W) from 29 children in 3 different schools. These children had received a single dose of ALB (400 mg). Prior treatment, before commencement of the study, may have occurred, but was not documented. Stool samples were preserved in 70% alcohol and stored at 4°C.

From another study carried out in Zanzibar and Uganda [9], we received 91 DNA samples from individual *A. lumbricoides* adult worms recovered after being expelled with “Combantrin” from patients who lived within areas where large scale interventions with benzimidazoles were ongoing. The DNA was preserved in alcohol and stored at 4°C.

### *A. lumbricoides* and *T. trichiura* Egg Recovery and DNA Extraction

The DNA assay was applied to eggs from non-treated (BZ-naïve subjects) and eggs from subjects in treated areas. *A. lumbricoides* eggs from Kenya were recovered from the uterus of adult female worms. Before the dissection of each female worm, the anterior end of the parasite was separated from the body to avoid contamination of adult DNA with DNA from eggs. Female worms were then opened longitudinally. At approximately one quarter length along the body, the uterus is attached to the genital pore and subsequently divides into two branches. Each uterine branch was cut and opened to release the eggs. From the study carried out in Panama, 29 pooled *A. lumbricoides* egg samples and 8 pooled *T. trichiura* egg samples were recovered from stool samples by using a flotation technique [10].

Following the recovery of eggs, DNA was extracted from pooled *A. lumbricoides* eggs collected from the uterus of adult female worms. It was also extracted from individual *A. lumbricoides* female and male worms and from individual *T. trichiura* adult worms using the DNeasy Blood and Tissue Extraction Kit (Qiagen) according to the manufacturer's protocol. For the DNA extraction of *A. lumbricoides* and *T. trichiura* eggs from stools samples from Panama, the QIAamp DNA stool mini kit (Qiagen) was used according to the manufacturer's protocol.

### Determination of the β-Tubulin Sequence in *A. lumbricoides* Adult Worms

Genomic sequence (GenBank AF034219) was available for *T. trichiura* β-tubulin, [11], but was not available for *A. lumbricoides*, so it was necessary to generate this sequence. Total RNA was extracted from adult *A. lumbricoides* from Kenya by TRIzol Reagent (Invitrogen Life Technologies, Burlington, ON) according to the manufacturer's protocol. Then, the total RNA was reverse transcribed with the oligo-dt (12–18) primer according to the manufacturer's instruction.

For the initial isolation of the *A. lumbricoides* β-tubulin gene, cDNA was amplified with degenerate primers. These primers were designed based on the conserved region of β-tubulin of six related nematodes: *Haemonchus contortus* (GenBank, M76493 - isotype 1), *Brugia malayi* (GenBank, AY705382), *Necator americanus* (GenBank, EF392851), *Trichuris trichiura* (GenBank, AF118385), *Teladorsagia circumcincta* (GenBank, Z69258 - isotype 1) and *Onchocerca volvulus* (GenBank, AF01886) (the alignments of β-tubulin cDNAs for 11 nematodes are given in Figure S1). Two sets of primers were designed for a nested PCR approach. In the first round PCR, the cDNA was amplified with the outer sense primer (5′-3′) CAAAGTGGAGCKGGHCACAACTGGC and the outer antisense primer (5′-3′) CGBAGATCHGCATTCAGCTGHCCAGG. The PCR product from the first round was then used as a template for the subsequent amplification using the nested primers, sense (5′-3′) CTYGGTGGAGGYACMGGWTC and antisense (5′-3′) CGBAGATCHGCATTCAGCTGHCCAGG. For both rounds, the PCR conditions were an initial denaturation at 95°C for 3 min, followed by 35 cycles of 95°C for 45 s, 60°C for 45 s, 72°C for 1 min and a final extension at 72°C for 5 min. Prior to cloning, a 5 µl aliquot of PCR product from the nested reaction was examined on a 1% agarose (TAE) electrophoresis gel to confirm the size of the product. The amplified PCR products were cloned into the pCR2.1 TOPO vector using a TOPO-TA-Cloning kit (Invitrogen, Life Technologies, Burlington, ON), as per the manufacturer's instructions and then sequenced.

Based on the sequenced fragment, gene-specific primers were designed for the 5′ and 3′ rapid amplification cDNA ends (RACE) reactions. For the 5′ RACE reaction two primers were designed, (5′-3′) CGTTGAGCGCCCTGTATGC and (5′-3′) CAACACCACATCAGAAACCT and used in a semi-nested PCR reaction with the nematode splice leader sequence SL1 (5′-3′) GGTTTAATTACCCAAGTTTGAG [12]. The amplification conditions for the primary reaction were 3 min at 94°C, followed by 40 cycles at 94°C for 45 s, 59.4°C for 45 s, 68°C for 1 min and a final extension at 68°C for 5 min. For the semi-nested reaction the amplification conditions were as outlined above with an annealing temperature of 55.8°C.

To isolate the 3′ end of β-tubulin cDNA, two gene specifics primers were designed (5′-3′) CCACGTCTTCACTTCTTCATG and (5′-3′) GTACGACATCTGCTTCAGGACCCTG, and used in a nested PCR reaction with oligo adaptor primers B_1_ (3231)(5′-3′) CCTCTGAAGGTTCACGGAT and B_2_ (3232)(5′-3′) CACGGATCCACATCTAGAT, respectively. The PCR conditions for both reactions were as described above with an annealing temperature of 60°C for the first reaction and 55.6°C for the nested reaction. The resulting fragments after the second round of each reaction were purified using the QIAGEN PCR purification kit according to the manufacturer's protocol. They were subsequently ligated into pGEM-T cloning vector (Promega, Madison, WI) and sequenced from both directions with SP6 and T7 vector primers. Phylogenetic analysis was undertaken with the UPGMA method and performed using the Mac vector program in order to identify the relationship between β-tubulin sequences of 13 nematodes.

### Development of Diagnostic Tests in *A. lumbricoides* and *T. trichiura* Benzimidazole Naïve Worms

To optimize the pyrosequencing DNA assay, two control plasmids were constructed for each species and contained either the “sensitive” codon TTC for the wild type or a mutation, generated by site-directed mutagenesis (TAC - mutant type), inserted at position 200. These plasmids were based on the amplification of a small portion of β-tubulin genomic sequence from *A. lumbricoides* and *T. trichiura* worms that were naïve for benzimidazole treatment. Gene-specific primers were designed to amplify a small fragment of genomic DNA (GenBank, FJ501301) surrounding the codon 200. In *A. lumbricoides*, primers were based on the sequence of the fragment of *A. lumbricoides* β-tubulin cDNA. All specific primers were designed with the software gene runner in the exonic region, sense (5′-3′) GGTGGAGGCACAGGATCTGGC, antisense (5′-3′) GCAGCCGCTCCTCG. For *T. trichiura*, primers were directly designed from the genomic sequence (GenBank AF034219), sense (5′-3′) GGTTTCAGATACAGTTGTAG (position 1212-1231, located 76 amino acids upstream of the first T of the codon 200 TTC) and antisense (5′-3′) CAAATGATTTAAGTCTCCG (position 1356–1374, located 146 amino acids upstream of the first T of the codon 200 TTC). PCR reaction conditions were an initial denaturation at 94°C for 3 min, followed by 35 cycles of 94°C for 45 s, an annealing temperature of 52°C in *A. lumbricoides* and 56°C in *T. trichiura*, for 45 s, and 68°C for 1 min and a final extension at 68°C for 5 min. Resulting fragments were cloned and sequenced as described above.

The site directed mutagenesis strategy consisted of the amplification of two overlapping fragments using outer and inner mutagenesis primer pairs. Then, the opposing PCR strands were annealed at overlapping regions, extended, and amplified by PCR to produce the desired full-length strand. Two pairs of primers were designed, in *A. lumbricoides* outer sense primers (5′-3′) GTTTCTGATGTGGTGTTGGAG, antisense (5′-3′) CAAATGGTTGAGGTCTCCG and inner mutagenesis primer, sense (5′-3′) CGATGAAACCTACTGCATTGACAATG, antisense (5′-3′) CAAATGGTTGAGGTCTCCG. The PCR conditions were as outlined above with an annealing temperature of 54°C. PCR products were electrophoresed though agarose gels and then purified. Ten µl (10–100 ng DNA) of each purified PCR product were mixed and denatured at 94°C for 3 min. The second PCR reaction had an annealing temperature of 53°C and allowed the production of the desired full-length strand. In *T. trichiura* the protocol used to generate the mutant type plasmid was as described above with outer sense (5′-3′) GGTTTCAGATACAGTTGTAG (position 1013–1031) and antisense (5′-3′) CAAATGATTTAAGTCTCCG (position 1356–1374) and inside mutagenesis primers, sense (5′-3′) CGGACGAAACATACTGCATAGATAATG, antisense (5′-3′) CATTATCTATGCAGTATGTTTCGTCCG (position 1277–1301). The annealing temperature for the first PCR was 54°C and 50°C for the second PCR. The fragments obtained with the desired mutation for both parasites were purified, cloned and subsequently sequenced.

### Genotyping by the Pyrosequencing Method

Pyrosequencing was used to detect a possible SNP in the genomic DNA from the field samples. First, a smaller fragment of DNA from the control plasmids that surrounded the position 200 was amplified. Subsequently, we amplified the same portion of β-tubulin DNA of eggs isolated from individual *A. lumbricoides* adult worms from Kenya, Zanzibar, Uganda and DNA from pools of eggs from Panama and Kenya. For *T. trichiura*, DNA of individual worms from Kenya and pooled eggs from Panama was also amplified. A fragment of 158 bp of *A. lumbricoides* β-tubulin DNA was amplified with primers: sense (5′-3′) AGGTTTCTGATGTGGTGTTGGA and antisense (5′-3′) TATGTGGGATTTGTAAGCTTCAG. For *T. trichiura*, a fragment of 163 bp was amplified with gene-specific primers: sense (5′-3′) AGGTTTCAGATACAGTTGTAG (position 1211–1231), antisense (5′-3′) CAAATGATTTAAGTCTCCG (position 1356–1374). The antisense primer was biotinylated (Invitrogen, Life technologies, Burlington, ON) at its 5′ end. For both reactions, the thermal cycling conditions included an initial incubation at 94°C for 3 min, followed by 50 cycles of 94°C for 45 s, an annealing temperature of 58.7°C for *A. lumbricoides*, 55°C for *T. trichiura*, 68°C for 1 min and a final extension at 68° C for 6 min. Biotinylated PCR products were immobilized on streptavidin-coated Sepharose beads (Amersham Biosciences, Piscataway, NJ) and sequencing primers used for SNP analysis in the PSQ96MA instrument (Biotage AB, Charlottesville, VA) were: (5′-3′) GAGAACACGGACGAAACAT (position 1270–1288) for *T. trichiura* and (5′-3′) GAGAACACCGATGAAACCT for *A. lumbricoides*.

### Confirmation of Genotype Sequences

Genotype sequences obtained by pyrosequencing were confirmed by conventional sequencing at the Quebec/McGill University Genome Centre.

## Results

### *A. lumbricoides* β-Tubulin cDNA Sequence

**Figure 1:**
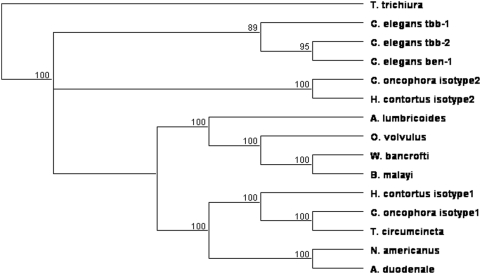
UPGMA tree showing the relationship between the β-tubulin cDNA sequences of *A. lumbricoides* with β-tubulin cDNA sequence of other nematodes. Method: UPGMA; bootstrap (100 reps); tie breaking = systematic. Distance: absolute (# differences). Gaps distributed proportionally.

Fresh RNA from *A. lumbricoides*, naïve for BZ-treatment, allowed us to generate a high quality partial length β-tubulin cDNA (GenBank, EU814697). The length of the portion sequenced was 1137 pb. The translation product revealed a putative sequence of 378 amino acids. The phylogenetic tree (Figure 1) showed that the *A. lumbricoides* sequence did not have a close relationship with the Strongylida order which includes the hookworms, *A. duodenale* and *N. americanus* as well as the veterinary nematodes *H. contortus* and *T. circumcincta*. It was also the case for the Trichocephalida which includes *T. trichiura*. In fact, the *A. lumbricoides* cDNA sequence seemed to be more closely related to the Spirudida order which includes the filarial nematodes.

### Design of Diagnostic Tests

**Figure 2:**
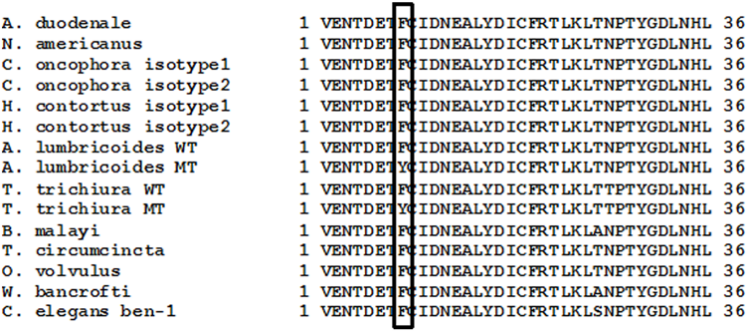
Alignment of *Ascaris lumbricoides* and *Trichuris trichiura* β-tubulin protein around codon 200, with related nematodes. WT = wild type, MT = mutant type. The square represents the amino acid at the position of interest.

Based on *A. lumbricoides* and *T. trichiura* β-tubulin, diagnostic SNP assays, optimized with control plasmids, were applied. An alignment of the portion around the codon of interest in the translated β-tubulin protein sequences of wild type (WT) and the mutant type (MT) plasmids with sequences of other nematodes highlighted a high conservation of the protein sequence. It also showed the (T→A) substitution (Phe200Tyr at the amino acid level, Figure 2) that is associated with BZ resistance in veterinary nematodes [13].

### β-Tubulin SNP Genotyping in Parasites from Untreated Subjects

Pyrosequencing assays were designed to genotype the single nucleotide polymorphism (SNP) at codon 200 of β-tubulin. This technique allows sequencing of short fragments of DNA in a very short period of time. The pyrosequencer used can process 96 samples in one hour, and the technique is highly accurate, making the results reliable and easy to interpret. This test was first evaluated with the help of the control plasmids. For *A. lumbricoides* and *T. trichiura*, we obtained distinct pyrogram profiles with the WT and MT plasmids. Pyrograms of each *Ascaris* and *Trichuris* control plasmid showed a single peak for the WT and MT plasmid that identified the “susceptible” TTC codon and the ‘resistant’ TAC, respectively, in the codon 200 position. Thus, these results confirmed that the diagnostic test was efficient and could easily and clearly identify the genotype of *A. lumbricoides* and *T. trichiura* worms for this position in the β-tubulin gene.

Once we obtained the expected genotype profiles, we applied the pyrosequencing diagnostic assay to *A. lumbricoides* adult worms and pooled eggs, and to *T. trichiura* adult worms from Kenya from people who had not received treatment with BZ drugs. For *A. lumbricoides* all individual females, males and pools of eggs were “sensitive” T/T. In contrast, we found more diversity in the *T. trichiura* population with regard to the (TAC) SNP. The frequency of heterozygote and homozygote worms was almost the same for male and female worms. Out of 20 males, 11 were homozygous T/T (55%), 8 were heterozygous T/A (40%) and 1 was homozygous A/A (5%). Among the 19 female worms, 11 were homozygous T/T (58%), and 8 were heterozygous T/A (42%).

### β-Tubulin Genotyping in Parasites Following Mass Drug Administration

In order to investigate if repeated exposure to BZ treatment had an impact on the polymorphism in β-tubulin at codon 200, associated with resistance in veterinary nematodes, we screened the β-tubulin genes of *A. lumbricoides* and *T. trichiura* from different areas where mass drug administration (MDA) programs had been in operation . Pyrosequencing assays, designed previously for each species were applied to detect the presence or absence of TTC or TAC at the SNP of interest. DNA from pooled eggs or DNA from individual worms was analysed. TTC and TAC at codon 200, in the parasite samples, were confirmed by conventional sequencing conducted at the Quebec/McGill University Genome Centre.

The genotype frequencies obtained for all species are shown in and are as follows:

- For *A. lumbricoides* from Uganda and Zanzibar all individual worms were T/T (100%)
- For *A. lumbricoides* from Panama, all the egg pools genotyped were only T (100%)
- For egg pools of *T. trichiura* from Panama, T and A, or A only, were found but no T only was found:
  - Out of 8 samples, 3 were T plus A (37%), and 5 were A only (63%).

## Discussion

The possible development of resistance to ALB and MBZ in human nematodes is a threat to MDA programs [8]. A number of studies have reported a reduced efficacy of ALB and MBZ anthelmintics in human STH after repeated treatments [9],[14]. Resistance in other nematodes is known to be associated with mutation in β-tubulin preventing the binding of BZ drugs [15]. Consequently, there is a need to investigate and monitor the frequency of SNPs in the β-tubulin gene [16],[17] that have been associated with BZ resistance. Bennett and co-workers used conventional sequencing on 72 individual *T. trichiura*, mostly from untreated individuals, and did not observe polymorphism at codon 200 in β-tubulin [18]. Using real time PCR and pyrosequencing, Schwab and colleagues have developed rapid genetic assays for individual *Wuchereria bancrofti* microfilaria [19]. Schwenkenbecher and co-workers have used real time PCR to assay for resistance SNPs in hookworms [20], but did not report finding the resistance associated SNP. However, the β-tubulin gene of *A. lumbricoides* had not been previously analysed. In this study, we developed pyrosequencing assays to detect SNPs in the β-tubulin genes of *A. lumbricoides* and *T. trichiura*, and analyzed samples obtained from naïve or benzimidazole treated individuals. Control plasmids, WT (TTC) and MT (TAC) were analyzed using the pyrosequencing method. The expected sequences, TTC (“sensitive”) and TAC (“resistant”) were obtained indicating that the assay was efficient. The development of these genetic assays allows a rapid screening method for the detection of possible resistance alleles in human parasites. This development is consistent with the aim of the Consortium for Anthelminthic Resistance SNPs (CARS) to develop panels of molecular markers for anthelmintic resistance in human and veterinary nematodes [15]. To date, there are not many reliable and accurate tools for diagnosing resistance in STH. Biological tests are used to assess resistance [21],[22] but have limited application as they generally can detect resistance only if the proportion of resistant worms is more than 25% [23]. The development of molecular assays for SNPs in the β-tubulin genes of STH gives new hope for monitoring for anthelmintic resistance. This is particularly important considering the difficulty in measuring resistance in helminth parasites of humans compared to helminth parasites of animals [24].

We genotyped STH species from Kenya before the implementation of MDA with ALB or MBZ in order to get baseline information on the SNP frequencies in the β-tubulin genes. In *A. lumbricoides* samples, heterozygous TTC/TAC or homozygous TAC/TAC were not found in samples from non-treated subjects. However, it would be interesting to do more sampling to confirm that the homozygous “sensitive” genotype predominates in different *A. lumbricoides* populations. It would also be interesting to analyse parasites from the same population of hosts after several rounds of treatment with ALB to see if repeated treatment over some years would result in the Tyr200 SNP being detected in *A. lumbricoides* worms.

In contrast, in *T. trichiura*, the SNP with TAC at codon 200 of β-tubulin was present in samples from the same Kenyan population. In *T. trichiura*, only one β-tubulin gene has been identified even with low stringency Southern blots [11]. This suggests that *T. trichiura* may carry only one isotype of the β-tubulin and that molecular change in this β-tubulin alone might result in resistance. In some veterinary nematodes, the sensitivity or resistance to BZ can be modulated by a second β-tubulin isotype [25]. Samples with both TTC and TAC, as well as others with TAC alone were identified in the *T. trichiura* population. Nevertheless, our sample size was low: out of 39 *T. trichiura* individual worms, 18 were heterozygous TTC/TAC and one individual was homozygous TAC/TAC. However, these frequencies may not be representative of a wider population of *T. trichiura*. Taking into account that the study was carried out in an area where MDA with BZs had not previously been implemented, the finding of a moderately high frequency of heterozygotes, as well as a low frequency of the “resistance” homozygote genotype, raises a concern should BZ deworming programs be implemented in the same region. However, even though the SNP was found as heterozygous in many parasites, a resistance phenotypic may not be apparent as the resistance may be recessive as has been reported for the Phe200Tyr SNP associated with resistance in *Teladorsagia circumcincta* [26]. This may delay the appearance of a resistance phenotype. However, further work will be necessary to correlate susceptibility/resistance phenotypes and genotypes to confirm the role of the codon 200 SNP with resistance in *T. trichiura* and to determine whether resistance is recessive, semi-dominant or dominant. However, repeated exposure with multiple rounds of treatment could lead to the loss of the susceptible allele as has already been demonstrated in veterinary nematodes [8],[27].

Even though the “resistance” (TAC) SNP was found in *T. trichiura* from MDA-naïve subjects, it is important to take into consideration that deworming programs are already common and have existed for many years in other regions of Kenya [28]–[30]. Communities or regions targeted are quite close to each other and people from treated areas could easily travel to non-treated areas [31].

It is interesting to note that many studies have demonstrated the lower efficacy of BZ drugs against *T. trichiura* compared with *A. lumbricoides* [9],[32],[33]. However, the factors involved in this difference of efficacy are not known, but could include pharmacokinetics, given the different locations of *A. lumbricoides* and *T. trichiura*, or differences in genetic predisposition to BZ susceptibility. Our current results could support the idea that the difference in sensitivity, between these species, could be due to the occurrence of different alleles with alternatively TTC or TAC at codon 200 in the β-tubulin gene within the gene pool of *T. trichiura*. In veterinary nematodes a number of factors including the frequency of anthelmintic use, proportion of the parasite population exposed to treatment, parasite turnover and other factors may contribute to the rate of development of resistance [34]. Implementation of very large scale control programs for STH and lymphatic filaria could increase drug selection pressure and possibly the frequency of the Tyr200 SNP in *T. trichiura*. Further analyses on the Phe200Tyr SNP in β-tubulin of *T. trichiura* worms and correlation of its frequency with benzimidazole efficacy will be important to determine so that the possible development of drug resistance as part of MDA programs for STH could be monitored by these assays. If the Tyr200 SNP is confirmed to be associated with BZ resistance in *T. trichiura*, it will also be important to investigate aspects of population dynamics which impact on the rate of change in SNP frequency in populations under drug pressure.

The final objective of our study was to determine if the frequency of the β-tubulin SNP varies after repeated treatment with BZ and if the resistance-associated SNP was high in *A. lumbricoides* and *T. trichiura* samples from areas where there had been MDA with ALB or MBZ. None of the *A. lumbricoides* samples from Panama, Uganda or Zanzibar, examined by pyrosequencing, carried the TAC mutation at codon 200 in the β-tubulin gene. However, in pooled egg samples of *T. trichiura* from Panama, we found the Phe200Tyr SNP in egg pools from hosts who were reported to have been treated with ALB. For the *T. trichiura* pooled egg samples from Panama, both mixed TTC/TAC and TAC alone, in different pools, were found. It is important to point out that as we used pooled eggs, we could not determine the frequency of different genotypes in the worm population. This means that SNP frequencies refer to between *T. trichiura* egg pools and not within a particular egg pool. Because of this, and the small number of samples, the SNP frequencies must be interpreted with caution. Based on the experience in veterinary nematodes where benzimidazole resistance appears to be recessive [26], a high frequency of the homozygous “resistance” genotype could affect the cure rate and the drug efficacy, and repeated treatment may increase the frequency of homozygous “resistance” genotypes and lead to a rapid development of drug resistance. A study carried out in South Africa on the drug efficacy of 400 mg ALB demonstrated a low cure rate for *T. trichiura* and the authors concluded that this drug was not appropriate for a deworming program in this region [35]. There is an urgent need for studies correlating drug efficacy with genotype.

Knowledge of the β-tubulin sequences will enable us to develop similar pyrosequencing assays for alternative SNPs in β-tubulin of STH, at codons 167 and 198, known to be involved in drug resistance in cyathostome nematodes [26],[36] and occasionally in *H. contortus* [7],[37].

Studies such as ours are required to help control program managers to make appropriate decisions for the design of treatment programs against these harmful parasites. In this study, we described a reliable, fast and easy DNA assay based on a pyrosequencing technique for two soil-transmitted nematodes of humans. This technique allows the SNP analysis of large numbers of egg samples or other parasite stages, in a short period of time. For the first time, we characterized the partial β-tubulin cDNA and genomic DNA sequence of *A. lumbricoides*. Knowledge of the β-tubulin sequences is important as little is known about resistance to benzimidazole drugs in human helminths and also because of the rapid development of drug resistance in veterinary nematodes. The SNP with TAC was found in individual worms of *T. trichiura* from non-treated people in Kenya, and in *T. trichiura* egg pools from treated people in Panama. These findings provide a possible explanation for the sometimes low efficacy of benzimidazole anthelmintics against *T. trichiura* and an important warning of the possibility that resistance may develop, particularly in *T. trichiura*. It is crucial to continue monitoring for the frequency of the codon 200 TTC/TAC SNP in areas under MDA and to confirm whether the TAC allele confers BZ resistance in these STHs.

## Supporting Information

Figure S1 Alignment of β-tubulin cDNA sequences from 11 nematodes.
(1.89 MB PDF)
