# Supplementary material for: Assays to Detect β-Tubulin Codon 200 Polymorphism in Trichuris trichiura and Ascaris lumbricoides
Source: PLoS Negl Trop Dis. 2009 Mar 24;3(3):e397. doi: 10.1371/journal.pntd.0000397 (PMC2654341; doi:10.1371/journal.pntd.0000397)
Supplement: Figure S1 — Alignment of β-tubulin cDNA sequences from 11 nematodes. (1.89 MB PDF) [file pntd.0000397.s001.pdf]

Alignment of beta tubulin cDNA sequences from 11 nematodes

```
                               10        20        30        40        50
C. oncophora isotype1                 GGTTTAATTACCCAAGTTGAGAACTCTCCTCGACTACA
C. oncophora isotype2  GGTTTAATTACCCAAGTTTGAGCCTTCTCCACAACCCACACCAATCCATCCATA
C. elegans tbb-1                           GCACACCCATTATTTCCTTCATTTTTCAGAAACA
C. elegans tbb-2                                          ATTACGACCTTTTCAGCAA
C. elegans ben-1
H. contortus isotype1                                      GAATTCCCGAGAACACAA
H. contortus isotype2                                              GAATTCCGCA
W. bancrofti
T. trichiura
T. circumcincta
O. volvulus
N. americanus
B. malayi
A. lumbricoides
A. duodenale

                           60        70        80        90       100
C. oncophora isotype1  TCATGCGTGAAATCGTTCATGTACAAGCCGGTCAATGCGGTAACCAGATTGGAT
C. oncophora isotype2  GAATGCGTGAGATCGTCCACGTTCAAGCCGGACAGTGCGGAAACCAGATCGGAG
C. elegans tbb-1       ACATGAGAGAAATCGTCCACGTTCAAGCCGGACAGTGCGGAAACCAGATCGGGT
C. elegans tbb-2       AAATGAGAGAGATCGTCCACGTGCAAGCCGGACAATGCGGAAACCAAATCGGAT
C. elegans ben-1         ATGAGAGAAATTGTTCACGTTCAAGCCGGACAATGTGGTAATCAAATCGGAG
H. contortus isotype1  TCATGCGTGAAATCGTTCATGTGCAAGCCGGTCAATGCGGCAACCAGATCGGAT
H. contortus isotype2  GAATGCGTGAGATCGTCCACGTTCAGGCCGGACAGTGCGGAAACCAGATCGGAG
W. bancrofti
T. trichiura             ATGCGTGAGATTGTGCATATCCAAGCCGGCCAATGTGGTAATCAAATTGGTG
T. circumcincta          ATGCGTGAAATCGTTCATGTACAAGCCGGTCAATGCGGTAACCAGATCGGAT
O. volvulus              ATGAGAGAAATTGTTCATGTTCAGGCTGGTCAATGTGGCAATCAAATTGGTG
N. americanus            ATGCGTGAGATTGTGCATGTGCAAGCTGGCCAATGTGGAAACCAGATCGGTT
B. malayi                ATGAGAGAAATTGTTCACGTTCAAGCTGGTCAATGTGGCAACCAGATTGGTG
A. lumbricoides
A. duodenale             ATGCGTGAGATCGTGCATGTACAAGCTGGCCAGTGTGGAAACCAGATCGGTT
```

```
                            110       120       130       140       150       160
C. oncophora isotype1       CAAAGTTCTGGGAAGTGATCTCCGATGAACACGGTATCCAACCCGATGGAACAT
C. oncophora isotype2       CAAAGTTCTGGGAGGTTATCTCTGACGAACACGGCATCCAGCCCGATGGATCAT
C. elegans tbb-1            CCAAGTTCTGGGAAGTCATCTCCGATGAGCACGGAATCCAGCCCGACGGAACCT
C. elegans tbb-2            CCAAATTCTGGGAGGTCATCTCCGACGAGCACGGAATCCAGCCAGACGGAACCT
C. elegans ben-1            CCAAGTTCTGGGAAGTGATATCCGATGAGCATGGGATCCAGCCTGATGGAACTT
H. contortus isotype1       CAAAGTTCTGGGAAGTGATCTCTGATGAGCACGGTATCCAGCCCGATGGAACAT
H. contortus isotype2       CCAAGTTCTGGGAGGTTATCTCTGACGAGCATGGCATCCAACCCGATGGATCAT
W. bancrofti
T. trichiura                CGAAGTTCTGGGAAGTCATCTCGGATGAACATGGTATCGATCCCACAGGAACTT
T. circumcincta             CAAAGTTCTGGGAAGTGATCTCTGATGAGCACGGTATCCAGCCCGATGGAACTT
O. volvulus                 CCAAGTTCTGGGAAGTAATATCGGATGAACATGGCATTCAACCTGATGGTACTT
N. americanus               CAAAGTTCTGGGAAGTGATCTCTGACGAACATGGTATCAAGCCTGATGGCACAT
B. malayi                   CCAAGTTCTGGGAAGTAATATCGGATGAACATGGTGTTCAACCTGATGGTACAT
A. lumbricoides
A. duodenale                CAAAGTTCTGGGAAGTGATCTCTGATGAGCACGGCATCAAGCCTGACGGCACCT

                                  170       180       190       200       210
C. oncophora isotype1       ACAAAGGAGAATCAGACCTGCAGTTAGAGAGAATCAATGTTTACTACAATGAAG
C. oncophora isotype2       ACAAAGGAGAAAGTGATCTTCAATTAGAACGAATTAATGTGTACTACAACGAAG
C. elegans tbb-1            TCAAGGGAGAAAGCGATCTCCAGCTGGAGAGAATCGATGTCTACTACAACGAGG
C. elegans tbb-2            TCAAGGGAGAGACGGATCTCCAGCTTGAGCGCATTGACGTCTACTACAACGAAG
C. elegans ben-1            ATAAGGGAGAAAGTGATTTGCAGTTGGAAAGAATCAATGTCTACTATAATGAGG
H. contortus isotype1       ACAAAGGAGAATCAGATCTGCAATTAGAAAGGATCAATGTGTACTACAATGAAG
H. contortus isotype2       ACAAAGGAGAGAGCGATCTACAACTAGAACGAATCAATGTTTACTACAATGAGG
W. bancrofti
T. trichiura                ACCACGGTGACTCGGACCTGCAACTTGAACGCATAAATGTTTACTACAACGAAG
T. circumcincta             ACAAAGGAGAATCGGCACTGCAATTAGAAAGAATCAATGTATACTACAATGAGG
O. volvulus                 ATAAAGGTGATTCAGATTTGCAAATTGAGCGAATCAATGTCTACTATAATGAAG
N. americanus               ACAGTGGCGAGTCTGATTTGCAGCTTGAGCGAATCAATGTATACTATAACGAAG
B. malayi                   ACAAAGGTGATTCAGACTTGCAAATTGAACGAATCAACGTCTACTATAATGAAG
A. lumbricoides
A. duodenale                ACAGTGGTGAATCTGATCTGCAACTTGAACGAATCAACGTGTACTATAATGAAG
```

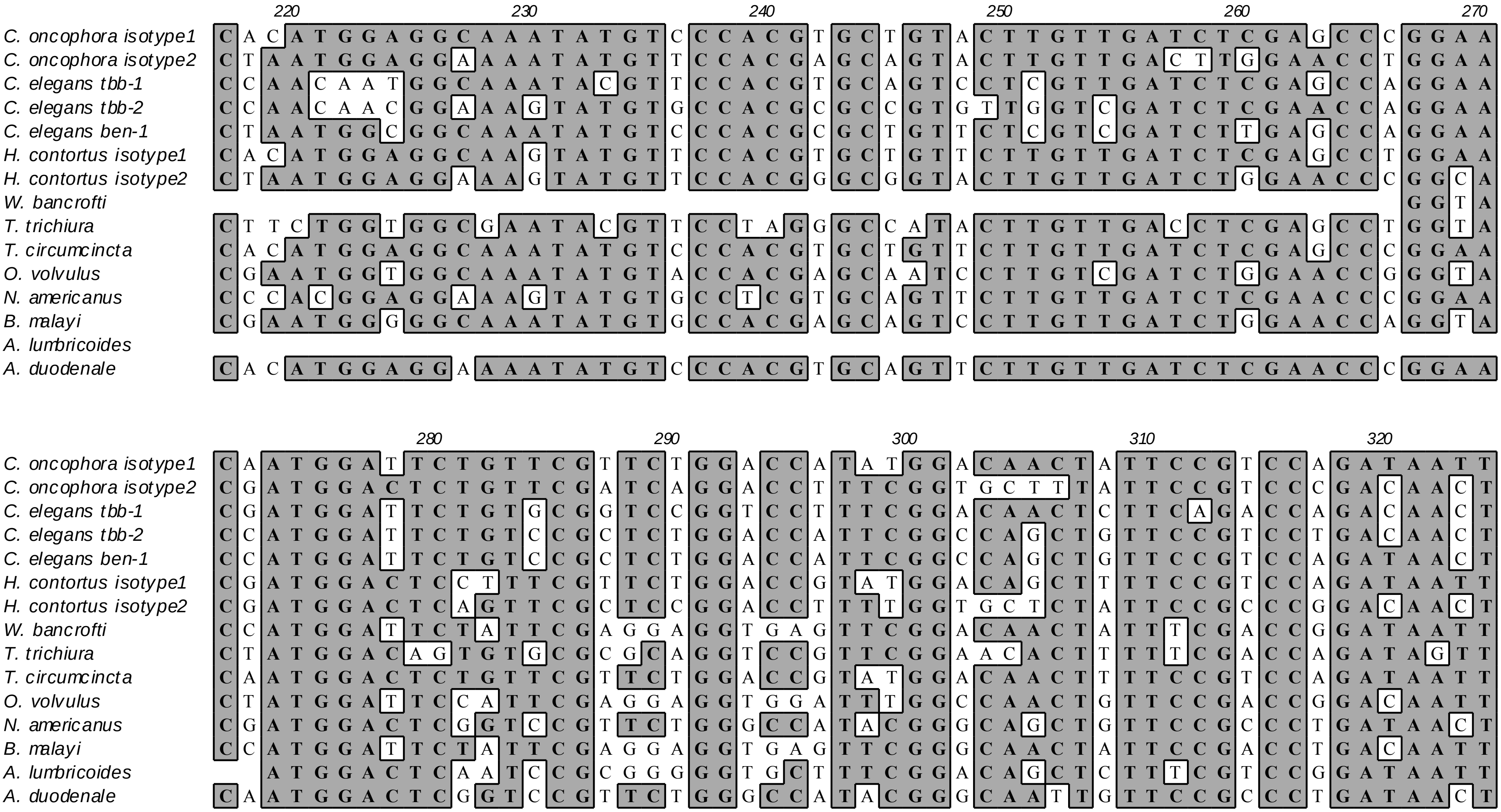
220 230 240 250 260 270
C. oncophora isotype1 C A C A T G G A G G C A A A T A T G T C C C A C G T G C T G T A C T T G T T G A T C T C G A G C C C G G A A
C. oncophora isotype2 C T A A T G G A G G A A A A T A T G T T C C A C G A G C A G T A C T T G T T G A C T T G G A A C C T G G A A
C. elegans tbb-1 C C A A C A A T G G C A A A T A C G T T C C A C G T G C A G T C C T C G T T G A T C T C G A G C C A G G A A
C. elegans tbb-2 C C A A C A A C G G A A A G T A T G T G C C A C G C G C C G T G T T G G T C G A T C T C G A A C C A G G A A
C. elegans ben-1 C T A A T G G C G G C A A A T A T G T C C C A C G C G C T G T T C T C G T C G A T C T T G A G C C A G G A A
H. contortus isotype1 C A C A T G G A G G C A A G T A T G T T C C A C G T G C T G T T C T T G T T G A T C T C G A G C C T G G A A
H. contortus isotype2 C T A A T G G A G G A A A G T A T G T T C C A C G G G C G G T A C T T G T T G A T C T G G A A C C C G G C A
W. bancrofti G G T A
T. trichiura C T T C T G G T G G C G A A T A C G T T C C T A G G G C C A T A C T T G T T G A C C T C G A G C C T G G T A
T. circumcincta C A C A T G G A G G C A A A T A T G T C C C A C G T G C T G T T C T T G T T G A T C T C G A G C C C G G A A
O. volvulus C G A A T G G T G G C A A A T A T G T A C C A C G A G C A A T C C T T G T C G A T C T G G A A C C G G G T A
N. americanus C C C A C G G A G G A A A G T A T G T G C C T C G T G C A G T T C T T G T T G A T C T C G A A C C C G G A A
B. malayi C G A A T G G G G G C A A A T A T G T G C C A C G A G C A G T C C T T G T T G A T C T G G A A C C A G G T A
A. lumbricoides
A. duodenale C A C A T G G A G G A A A A T A T G T C C C A C G T G C A G T T C T T G T T G A T C T C G A A C C C G G A A
280 290 300 310 320
C. oncophora isotype1 C A A T G G A T T C T G T T C G T T C T G G A C C A T A T G G A C A A C T A T T C C G T C C A G A T A A T T
C. oncophora isotype2 C G A T G G A C T C T G T T C G A T C A G G A C C T T T C G G T G C T T T A T T C C G T C C C G A C A A C T
C. elegans tbb-1 C G A T G G A T T C T G T G C G G T C C G G T C C T T T C G G A C A A C T C T T C A G A C C A G A C A A C T
C. elegans tbb-2 C C A T G G A T T C T G T C C G C T C T G G A C C A T T C G G C C A G C T G T T C C G T C C T G A C A A C T
C. elegans ben-1 C C A T G G A T T C T G T C C G C T C T G G A C C A T T C G G C C A G C T G T T C C G T C C A G A T A A C T
H. contortus isotype1 C G A T G G A C T C C T T T C G T T C T G G A C C G T A T G G A C A G C T T T T C C G T C C A G A T A A T T
H. contortus isotype2 C G A T G G A C T C A G T T C G C T C C G G A C C T T T T G G T G C T C T A T T C C G C C C G G A C A A C T
W. bancrofti C C A T G G A T T C T A T T C G A G G A G G T G A G T T C G G A C A A C T A T T T C G A C C G G A T A A T T
T. trichiura C T A T G G A C A G T G T G C G C G C A G G T C C G T T C G G A A C A C T T T T T C G A C C A G A T A G T T
T. circumcincta C A A T G G A C T C T G T T C G T T C T G G A C C G T A T G G A C A A C T T T T C C G T C C A G A T A A T T
O. volvulus C T A T G G A T T C C A T T C G A G G A G G T G G A T T T G G C C A A C T G T T C C G A C C G G A C A A T T
N. americanus C G A T G G A C T C G G T C C G T T C T G G G C C A T A C G G G C A G C T G T T C C G C C C T G A T A A C T
B. malayi C C A T G G A T T C T A T T C G A G G A G G T G A G T T C G G G C A A C T A T T C C G A C C T G A C A A T T
A. lumbricoides A T G G A C T C A A T C C G C G G G G G T G C T T T C G G A C A G C T C T T T C G T C C G G A T A A T T
A. duodenale C A A T G G A C T C G G T C C G T C T G G G C C A T A C G G G C A A T T G T T C C G C C C T G A T A A C T

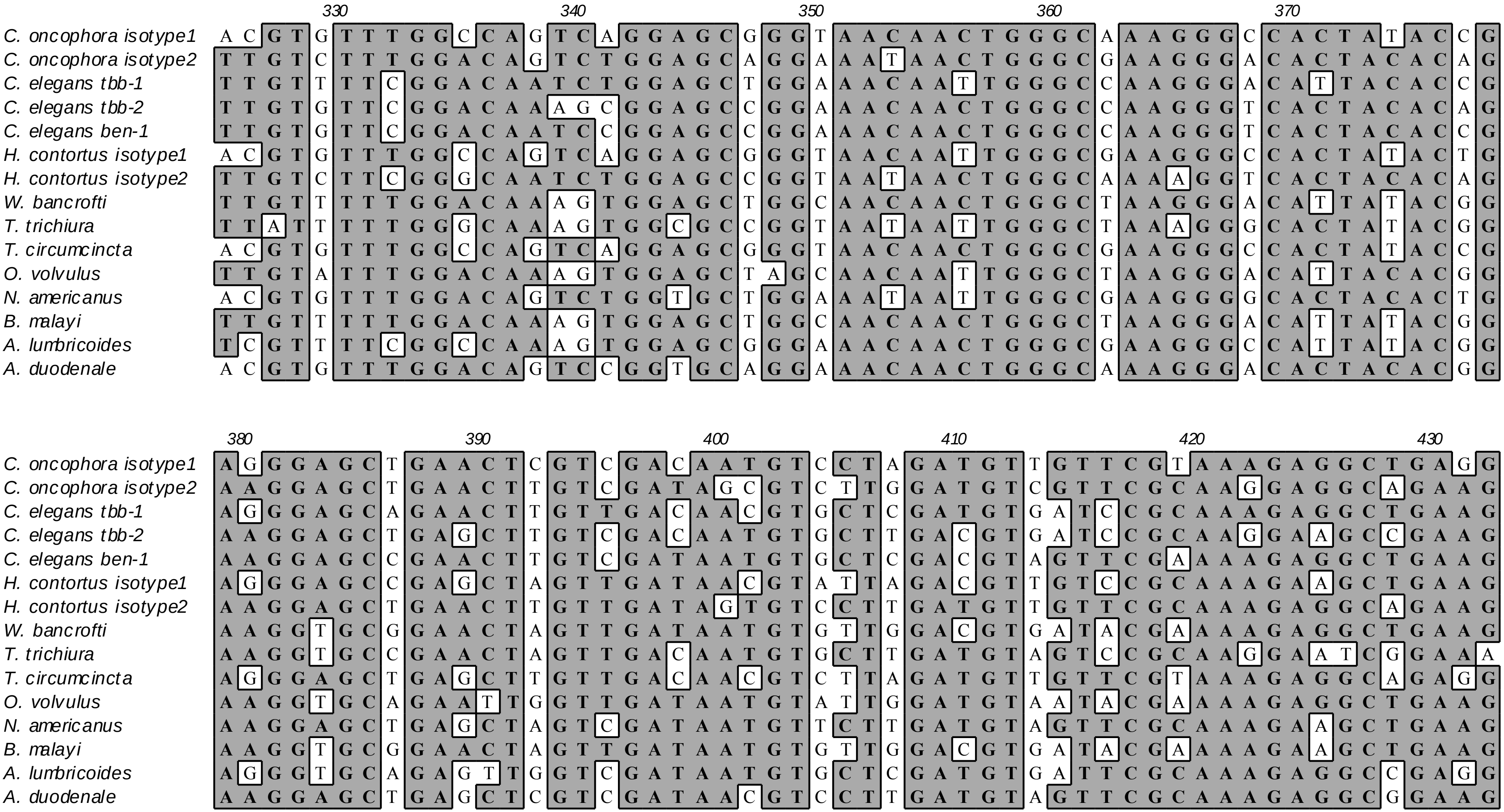
330 340 350 360 370
C. oncophora isotype1 ACGTGTTTGGCCAGTCAGGAGCGGGTAACAACTGGGCAAAGGGCCACTATACCG
C. oncophora isotype2 TTGTCTTTGGACAGTCTGGAGCAGGAAATAACTGGGCGAAGGGACACTACACAG
C. elegans tbb-1 TTGTTTTCGGACAATCTGGAGCTGGAAACAATTGGGCCAAGGGACATTACACCG
C. elegans tbb-2 TTGTGTTCGGACAAAGCGGAGCCGGAAACAACTGGGCCAAGGGTCACTACACAG
C. elegans ben-1 TTGTGTTCGGACAATCCGGAGCCGGAAACAACTGGGCCAAGGGTCACTACACCG
H. contortus isotype1 ACGTGTTTGGCCAGTCAGGAGCGGGTAACAATTGGGCGAAGGGCCACTATACTG
H. contortus isotype2 TTGTCTTCGGGCAATCTGGAGCCGGTAATAACTGGGCAAAAGGTCACTACACAG
W. bancrofti TTGTTTTTGGACAAAGTGGAGCTGGCAACAACTGGGCTAAGGGACATTATACGG
T. trichiura TTATTTTTGGGCAAAGTGGCGCCGGTAATAATTGGGCTAAAGGGCACTATACGG
T. circumcincta ACGTGTTTGGCCAGTCAGGAGCGGGTAACAACTGGGCGAAGGGCCACTATACCG
O. volvulus TTGTATTTGGACAAAGTGGAGCTAGCAACAATTGGGCTAAGGGACATTACACGG
N. americanus ACGTGTTTGGACAGTCTGGTGCTGGAAATAATTGGGCGAAGGGGCACTACACTG
B. malayi TTGTTTTTGGACAAAGTGGAGCTGGCAACAACTGGGCTAAGGGACATTATACGG
A. lumbricoides TCGTTTTCGGCCAAAGTGGAGCGGGAAACAACTGGGCGAAGGGCCATTATACGG
A. duodenale ACGTGTTTGGACAGTCCGGTGCAGGAAACAACTGGGCAAAGGGACACTACACGG
380 390 400 410 420 430
C. oncophora isotype1 AGGGAGCTGAACTCGTCGACAATGTCCTAGATGTTGTTCGTAAAGAGGCTGAGG
C. oncophora isotype2 AAGGAGCTGAACTTGTCGATAGCGTCTTGGATGTCGTTCGCAAGGAGGCAGAAG
C. elegans tbb-1 AGGGAGCAGAACTTGTTGACAACGTGCTCGATGTGATCCGCAAAGAGGCTGAAG
C. elegans tbb-2 AAGGAGCTGAGCTTGTCGACAATGTGCTTGACGTGATCCGCAAGGAAGCCGAAG
C. elegans ben-1 AAGGAGCCGAACTTGTCGATAATGTGCTCGACGTAGTTCGAAAAGAGGCTGAAG
H. contortus isotype1 AGGGAGCCGAGCTAGTTGATAACGTATTAGACGTTGTCCGCAAAGAAGCTGAAG
H. contortus isotype2 AAGGAGCTGAACTTGTTGATAGTGTCCTTGATGTTGTTCGCAAAGAGGCAGAAG
W. bancrofti AAGGTGCGGAACTAGTTGATAATGTGTTGGACGTGATACGAAAAGAGGCTGAAG
T. trichiura AAGGTGCCGAACTAGTTGACAATGTGCTTGATGTAGTCCGCAGGAATCGGAAA
T. circumcincta AGGGAGCTGAGCTTGTTGACAACGTCTTAGATGTTGTTCGTAAAGAGGCAGAGG
O. volvulus AAGGTGCAGAATTGGTTGATAATGTATTGGATGTAATACGAAAAGAGGCTGAAG
N. americanus AAGGAGCTGAGCTAGTCGATAATGTTCTTGATGTAGTTCGCAAAGAAGCTGAAG
B. malayi AAGGTGCGGAACTAGTTGATAATGTGTTGGACGTGATACGAAAAGAAGCTGAAG
A. lumbricoides AGGGTGCAGAGTTGGTCGATAATGTGCTCGATGTGATTCGCAAAGAGGCCGAGG
A. duodenale AAGGAGCTGAGCTCGTCGATAACGTCCTTGATGTAGTTCGCAAAGAGGCGGAAG

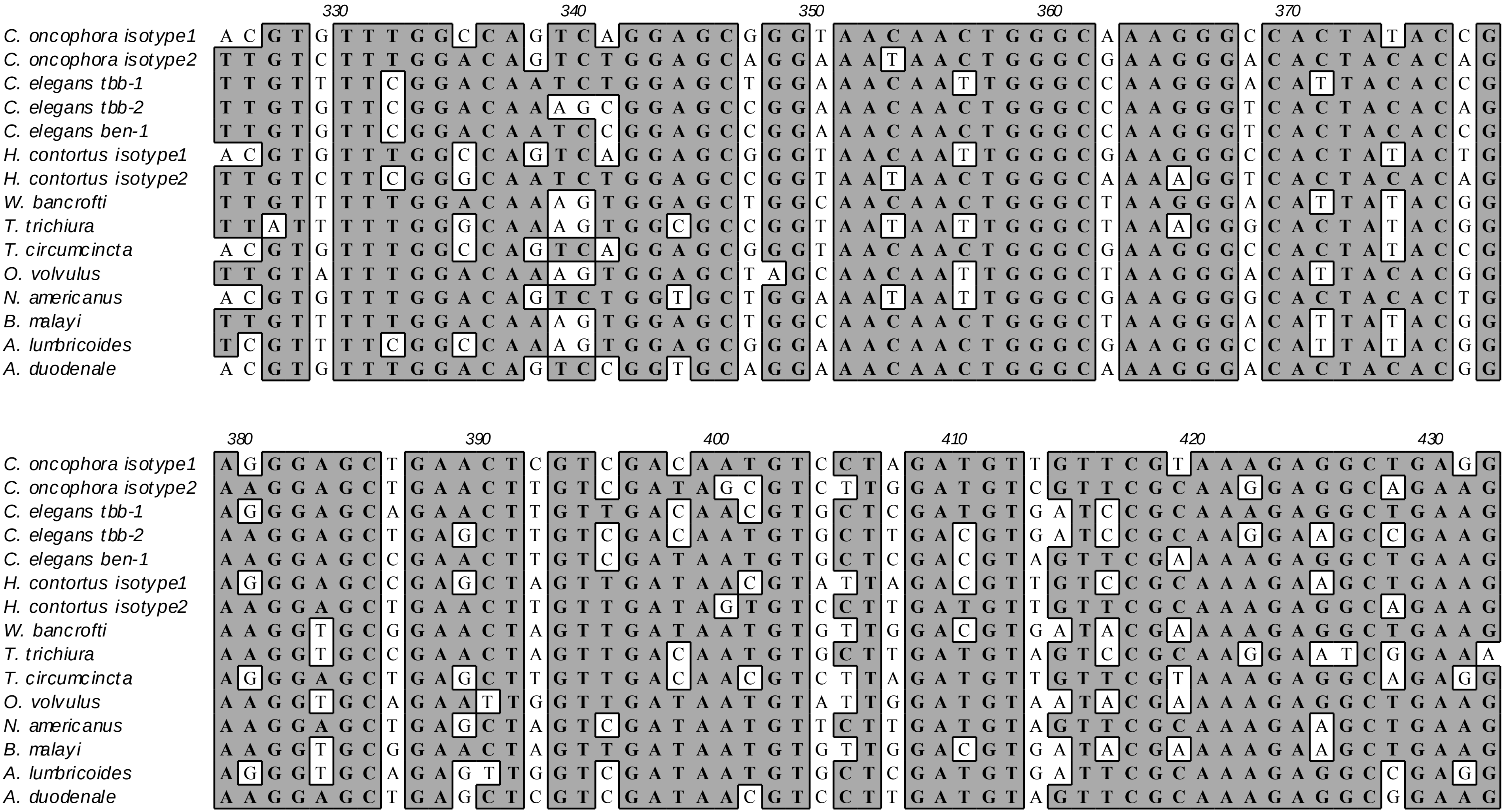
330 340 350 360 370
C. oncophora isotype1 ACGTGTTTGGCCAGTCAGGAGCGGGTAACAACTGGGCAAAGGGCCACTATACCG
C. oncophora isotype2 TTGTCTTTGGACAGTCTGGAGCAGGAAATAACTGGGCGAAGGGACACTACACAG
C. elegans tbb-1 TTGTTTTCGGACAATCTGGAGCTGGAAACAATTGGGCCAAGGGACATTACACCG
C. elegans tbb-2 TTGTGTTCGGACAAAGCGGAGCCGGAAACAACTGGGCCAAGGGTCACTACACAG
C. elegans ben-1 TTGTGTTCGGACAATCCGGAGCCGGAAACAACTGGGCCAAGGGTCACTACACCG
H. contortus isotype1 ACGTGTTTGGCCAGTCAGGAGCGGGTAACAATTGGGCGAAGGGCCACTATACTG
H. contortus isotype2 TTGTCTTCGGGCAATCTGGAGCCGGTAATAACTGGGCAAAAGGTCACTACACAG
W. bancrofti TTGTTTTTGGACAAAGTGGAGCTGGCAACAACTGGGCTAAGGGACATTATACGG
T. trichiura TTATTTTTGGGCAAAGTGGCGCCGGTAATAATTGGGCTAAAGGGCACTATACGG
T. circumcincta ACGTGTTTGGCCAGTCAGGAGCGGGTAACAACTGGGCGAAGGGCCACTATACCG
O. volvulus TTGTATTTGGACAAAGTGGAGCTAGCAACAATTGGGCTAAGGGACATTACACGG
N. americanus ACGTGTTTGGACAGTCTGGTGCTGGAAATAATTGGGCGAAGGGGCACTACACTG
B. malayi TTGTTTTTGGACAAAGTGGAGCTGGCAACAACTGGGCTAAGGGACATTATACGG
A. lumbricoides TCGTTTTCGGCCAAAGTGGAGCGGGAAACAACTGGGCGAAGGGCCATTATACGG
A. duodenale ACGTGTTTGGACAGTCCGGTGCAGGAAACAACTGGGCAAAGGGACACTACACGG
380 390 400 410 420 430
C. oncophora isotype1 AGGGAGCTGAACTCGTCGACAATGTCCTAGATGTTGTTCGTAAAGAGGCTGAGG
C. oncophora isotype2 AAGGAGCTGAACTTGTCGATAGCGTCTTGGATGTCGTTCGCAAGGAGGCAGAAG
C. elegans tbb-1 AGGGAGCAGAACTTGTTGACAACGTGCTCGATGTGATCCGCAAAGAGGCTGAAG
C. elegans tbb-2 AAGGAGCTGAGCTTGTCGACAATGTGCTTGACGTGATCCGCAGGAAGCCGAAG
C. elegans ben-1 AAGGAGCCGAACTTGTCGATAATGTGCTCGACGTAGTTCGAAAAGAGGCTGAAG
H. contortus isotype1 AGGGAGCCGAGCTAGTTGATAACGTATTAGACGTTGTCCGCAAAGAAGCTGAAG
H. contortus isotype2 AAGGAGCTGAACTTGTTGATAGTGTCCTTGATGTTGTTCGCAAAGAGGCAGAAG
W. bancrofti AAGGTGCGGAACTAGTTGATAATGTGTTGGACGTGATACGAAAAGAGGCTGAAG
T. trichiura AAGGTGCCGAACTAGTTGACAATGTGCTTGATGTAGTCCGCAGGAATCGGAAA
T. circumcincta AGGGAGCTGAGCTTGTTGACAACGTCTTAGATGTTGTTCGTAAAGAGGCAGAGG
O. volvulus AAGGTGCAGAATTGGTTGATAATGTATTGGATGTAATACGAAAAGAGGCTGAAG
N. americanus AAGGAGCTGAGCTAGTCGATAATGTTCTTGATGTAGTTCGCAAAGAAGCTGAAG
B. malayi AAGGTGCGGAACTAGTTGATAATGTGTTGGACGTGATACGAAAAGAAGCTGAAG
A. lumbricoides AGGGTGCAGAGTTGGTCGATAATGTGCTCGATGTGATTCGCAAAGAGGCCGAGG
A. duodenale AAGGAGCTGAGCTCGTCGATAACGTCCTTGATGTAGTTCGCAAAGAGGCGGAAG

```
                              440       450       460       470       480
C. oncophora isotype1   GCTGCGATTGCCTTCAGGGCTTCCAACTTACGCATTCTCTTGGAGGAGGTACCG
C. oncophora isotype2   GATGCGATTGCCTTCAGGGTTTCCCACTCACGCACTCGCTTGGAGGAGGCACTG
C. elegans tbb-1        GATGCGATTGTCTTCAAGGATTCCAGCTCACACACTCTCTTGGAGGTGGAACCG
C. elegans tbb-2        GATGCGATTGTCTTCAAGGATTCCAACTCACTCACTCTCTCGGAGGAGGAACCG
C. elegans ben-1        GATGTGATTGTCTTCAAGGATTCCAGTTGACTCATTCACTTGGAGGAGGAACTG
H. contortus isotype1   GTTGTGATTGCCTTCAGGGCTTCCAATTGACGCATTCACTTGGAGGAGGCACTG
H. contortus isotype2   GGTGCGATTGTCTTCAGGGTTTCCAACTCACGCACTCGCTTGGAGGAGGCACAG
W. bancrofti            GATGCGATTGTCTTCAGGGATTTCAACTAACGCATTCACTTGGTGGCGGTACCG
T. trichiura            GCTGCGACTGCCTGCAAGGGTTTCAGTTGACTCATTCCCTCGGCGGCGGAACTG
T. circumcincta         GTTGCGATTGCCTTCAGGGCTTCCAACTGACGCATTCTTTGGGAGGAGGTACTG
O. volvulus             GATGCGACTGTCTTCAGGGATTTCAATTGACTCATTCACTTGGCGGTGGTACCG
N. americanus           GATGTGACTGTCTCCAGGGCTTCCAATTGACCCACTCTCTTGGAGGAGGTACCG
B. malayi               GATGCGATTGTCTTCAGGGATTTCAACTAACGCATTCACTTGGTGGTGGTACCG
A. lumbricoides         GTTGCGACTGCCTCCAGGGTTTCCAGCTGACGCACTCGCTTGGTGGAGGCACAG
A. duodenale            GATGCGACTGTCTTCAGGGCTTCCAATTGACTCACTCACTTGGAGGAGGTACCG

                           490       500       510       520       530       540
C. oncophora isotype1   GATCCGGTATGGGCACTTTGCTTATTTCAAAAATTCGTGAGGAGTACCCTGACA
C. oncophora isotype2   GATCAGGAATGGGAACTTTATTGATTGCTAAGATCCGTGAAGAGTACCCTGATA
C. elegans tbb-1        GATCCGGAATGGGAACCCTTCTCATCTCCAAGATTCGCGAGGAATTCCCAGATC
C. elegans tbb-2        GATCTGGAATGGGAACACTTCTCATCTCCAAAATCCGCGAAGAGTACCCAGACA
C. elegans ben-1        GATCTGGAATGGGAACTCTTCTCATTTCGAAAATCCGTGAAGAGTATCCAGATA
H. contortus isotype1   GATCTGGAATGGGCACTTTGTTAATTTCAAAAATTCGTGAAGAGTACCCTGATA
H. contortus isotype2   GATCAGGAATGGGAACTCTGTTGATTGCTAAGATCCGTGAAGAATATCCTGATA
W. bancrofti            GTTCCGGTATGGGAACATTGCTGATCTCGAAAATCCGTGAGGAGTATCCGGATC
T. trichiura            GGAGTGGAATGGGTACGCTTCTGATATCTAAAATTCGGGAAGAGTATCCTGACC
T. circumcincta         GTTCGGGTATGGGCACTTTGCTCATCTCCAAAATTCGCGAGGAGTATCCGGATA
O. volvulus             GTTCTGGTATGGGAACATTGTTGATCTCGAAAATTCGTGAGGAATATCCGGATC
N. americanus           GATCAGGAATGGGAACACTTCTTATTTCTAAAATTCGGGAAGAATACCCTGATA
B. malayi               GTTCCGGCATGGGAACATTGCTGATCTCGAAAATTCGTGAGGAGTATCCAGATC
A. lumbricoides         GATCTGGCATGGGAACGTTGCTTATTTCGAAGATCCGTGAAGAATACCCCGACA
A. duodenale            GATCGGGTATGGGAACTCTCCTCATCTCTAAAATCCGCGAGGAGTACCCTGATA
```

550 560 570 580 590

C. oncophora isotype1	GAATTATGGCTTCGTTCTCTGTTGTTCCTTCACCAAAGGTCTCCGACACCGTTG
C. oncophora isotype2	GGATCATGTCTTCTTTCTCAGTGGTGCCTTCACCCAAGGTTTCCGATACCGTAG
C. elegans tbb-1	GAATTATGTCTTCCTTCTCGGTTGTTCCATCACCAAAGGTTTCGGATACCGTCG
C. elegans tbb-2	GAATCATGAGCTCATTCTCGGTTGTACCATCGCCAAAGGTGTCCGACACAGTTG
C. elegans ben-1	GAATTATGAGTTCTTTCTCGGTTGTTCCGTCGCCAAAGGTCTCGGACACAGTCG
H. contortus isotype1	GAATTATGGCTTCGTTCTCCGTTGTTCCATCACCCAAGGTATCCGACACTGTCG
H. contortus isotype2	GAATTATGTCATCCTTCTCAGTGGTACCTTCACCAAAGGTTTCCGATACTGTCG
W. bancrofti	GAATTATGAGCTCTTTTTCGGTTGTGCCGTCGCCCAAAGTATCAGATGTTGTGT
T. trichiura	GAATTATGACAACTTTTAGTGTCGTTCCGTCTCCGAAGGTTTCAGATACAGTTG
T. circumcincta	GAATCATGGCTTCATTCTCCGTTGTTCCATCACCTAAGGTATCCGACACCGTTG
O. volvulus	GAATTATGAGCTCTTTTTCGGTTGTACCATCACCCAAAGTATCAGATGTTGTAT
N. americanus	GAATCATGTCCTCGTTTTCCGTTGTACCCTCGCCAAAGGTTTCCGACACTGTGG
B. malayi	GAATTATGAGCTCTTTTTCGGTTGTGCCATCGCCCAAAGTATCAGATGTTGTGT
A. lumbricoides	GAATCATGAGCTCGTTCTCAGTTGTTCCATCTCCAAAGGTTTCTGATGTGGTGT
A. duodenale	GGATTATGTCCTCGTTCTCTGTTGTCCCCTCACCAAAGGTTTCCGACACCGTGG

600 610 620 630 640

C. oncophora isotype1	TGGAACCTTACAATGCTACTCTTTCTGTTCACCAACTGGTAGAAAATACCGATG
C. oncophora isotype2	TTGAACCTTACAATGCCACGCTTTCTGTGCACCAACTTGTGGAGAACACCGATG
C. elegans tbb-1	TCGAGCCGTACAATGCCACCCTCTCTGTCCACCAGCTTGTTGAGAACACCGACG
C. elegans tbb-2	TAGAACCATACAATGCCACCCTCTCCGTCCACCAGCTTGTTGAGAACACCGATG
C. elegans ben-1	TCGAGCCATACAACGCTACTCTTTCTGTCCACCAGCTCGTTGAAAATACCGATG
H. contortus isotype1	TAGAACCCTACAATGCTACCCTTTCCGTCCATCAACTGGTAGAGAACACCGATG
H. contortus isotype2	TGGAACCTTACAATGCCACCCTATCTGTCCATCAACTGGTGGAAAATACTGACG
W. bancrofti	TGGAACCCTACAATGCAACATTATCAGTCCACCAACTAGTTGAAAACACTGACG
T. trichiura	TAGAACCATATAATGCAACTCTGTCAGTCCACCAGTTGGTAGAGAACACGGACG
T. circumcincta	TGGAACCTTACAATGCCACTCTTTCTGTACACCAATTGGTAGAAAACACCGATG
O. volvulus	TGGAACCTTATAACGCAACATTATCAGTGCATCAATTAGTTGAAAACACTGACG
N. americanus	TTGAGCCATACAATGCTACACTCTCTGTTCACCAGTTAGTTGAGAATACAGATG
B. malayi	TGGAACCCTACAATGCAACATTATCAGTCCACCAACTAGTTGAAAACACTGACG
A. lumbricoides	TGGAGCCTTACAACGCAACGTTGTCCGTTCATCAGTTGGTTGAGAACACCGATG
A. duodenale	TGGAGCCATACAATGCTACACTCTCTGTGCACCAGCTGGTTGAGAATACAGATG

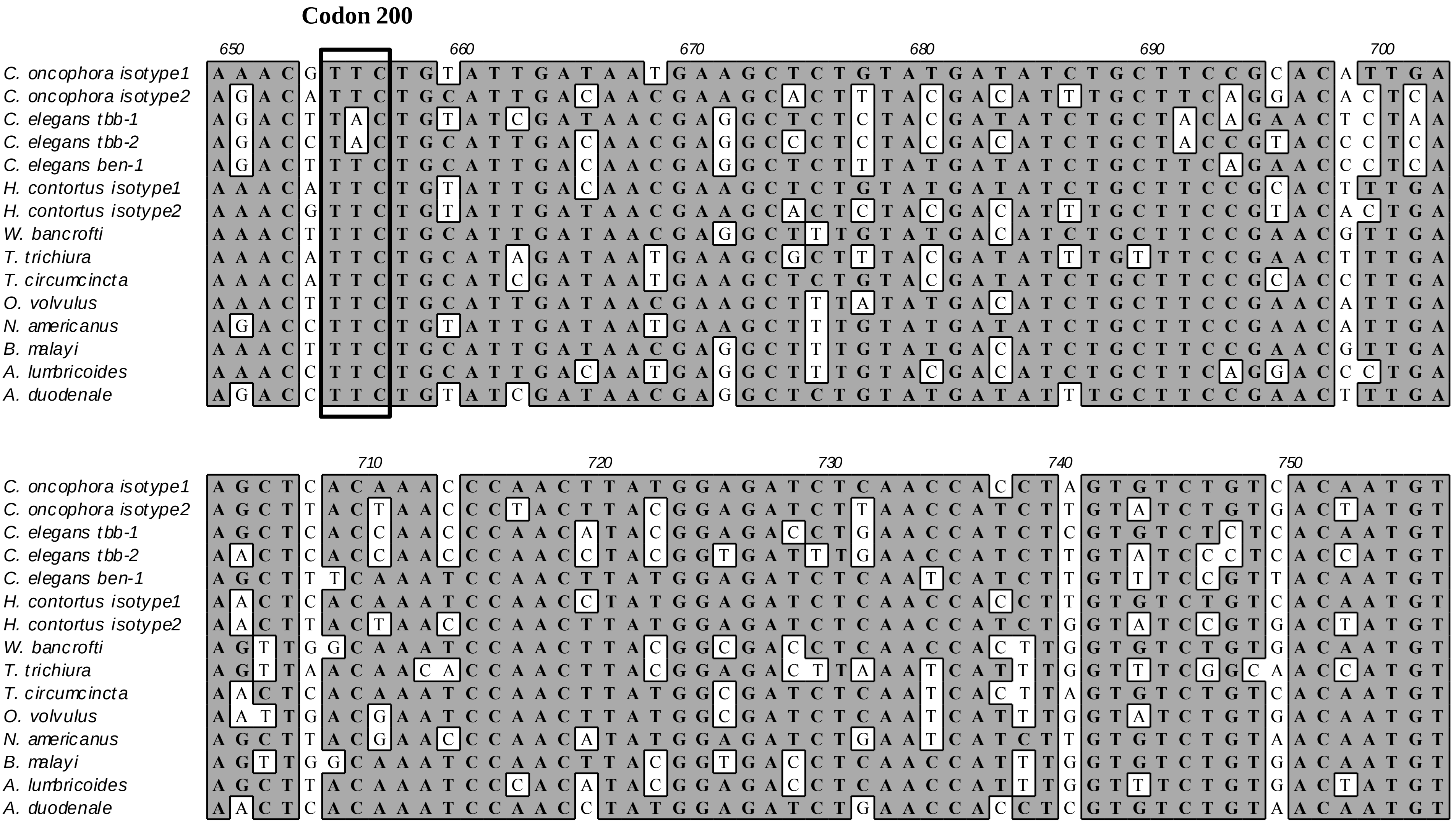
Codon 200
650 660 670 680 690 700
C. oncophora isotype1 AAACGTTCTGTATTGATAATGAAGCTCTGTATGATATCTGCTTCCGCACATTGA
C. oncophora isotype2 AGACATTCTGCATTGACAACGAAGCACTTTACGACATTTGCTTCAGGACACTCA
C. elegans tbb-1 AGACTTACTGTATCGATAACGAGGCTCTCTACGATATCTGCTACAGAACTCTAA
C. elegans tbb-2 AGACCTACTGCATTGACAACGAGGCCCTCTACGACATCTGCTACCGTACCCTCA
C. elegans ben-1 AGACTTTCTGCATTGACAACGAGGCTCTTTATGATATCTGCTTCAGAACCCTCA
H. contortus isotype1 AAACATTCTGTATTGACAACGAAGCTCTGTATGATATCTGCTTCCGCACTTTGA
H. contortus isotype2 AAACGTTCTGTATTGATAACGAAGCACTCTACGACATTTGCTTCCGTACACTGA
W. bancrofti AAACTTTCTGCATTGATAACGAGGCTTTGTATGACATCTGCTTCCGAACGTTGA
T. trichiura AAACATTCTGCATAGATAATGAAGCGCTTTACGATATTTGTTTCCGAACTTTGA
T. circumcincta AAACATTCTGCATCGATAATGAAGCTCTGTACGATATCTGCTTCCGCACCTTGA
O. volvulus AAACTTTCTGCATTGATAACGAAGCTTTATATGACATCTGCTTCCGAACATTGA
N. americanus AGACCTTCTGTATTGATAATGAAGCTTTGTATGATATCTGCTTCCGAACATTGA
B. malayi AAACTTTCTGCATTGATAACGAGGCTTTGTATGACATCTGCTTCCGAACGTTGA
A. lumbricoides AAACCTTCTGCATTGACAATGAGGCTTTGTACGACATCTGCTTCAGGACCCTGA
A. duodenale AGACCTTCTGTATCGATAACGAGGCTCTGTATGATATTTGCTTCCGAACTTTGA
710 720 730 740 750
C. oncophora isotype1 AGCTCACAAACCCAACTTATGGAGATCTCAACCACCTAGTGTCTGTCACAATGT
C. oncophora isotype2 AGCTTACTAACCCTACTTACGGAGATCTTAACCATCTTGTATCTGTGACTATGT
C. elegans tbb-1 AGCTCACCAACCCAACATACGGAGACCTGAACCATCTCGTGTCTCTCACAATGT
C. elegans tbb-2 AACTCACCAACCCAACCTACGGTGATTGAACCATCTTGTATCCCTCACCATGT
C. elegans ben-1 AGCTTTCAAATCCAACTTATGGAGATCTCAATCATCTTGTTTCCGTTACAATGT
H. contortus isotype1 AACTCACAAATCCAACCTATGGAGATCTCAACCACCTTGTGTCTGTCACAATGT
H. contortus isotype2 AACTTACTAACCCAACTTATGGAGATCTCAACCATCTGGTATCCGTGACTATGT
W. bancrofti AGTTGGCAAATCCAACTTACGGCGACCTCAACCACTTGGTGTCTGTGACAATGT
T. trichiura AGTTAACAACACCAACTTACGGAGACTTAAATCATTTGGTTTCGGCAACCATGT
T. circumcincta AACTCACAAATCCAACTTATGGCGATCTCAATCACTTAGTGTCTGTCACAATGT
O. volvulus AATTGACGAATCCAACTTATGGCGATCTCAATCATTTGGTATCTGTGACAATGT
N. americanus AGCTTACGAACCCAACATATGGAGATCTGAATCATCTTGTGTCTGTAACAATGT
B. malayi AGTTGGCAAATCCAACTTACGGTGACCTCAACCATTTGGTGTCTGTGACAATGT
A. lumbricoides AGCTTACAAATCCCACATACGGAGACCTCAACCATTTGGTTTCTGTGACTATGT
A. duodenale AACTCACAAATCCAACCTATGGAGATCTGAACCACCTCGTGTCTGTAACAATGT

	760 – 810
C. oncophora isotype1	CCGGAGTCACGACTTGCCTCCGATTCCCTGGACAACTAAATGCTGATCTTCGCA
C. oncophora isotype2	CTGGAGTAACAACGTGCTTACGTTTCCCTGGACAACTGAATGCTGATCTACGTA
C. elegans tbb-1	CTGGAGTGACTACCTGCCTCCGCTTCCCTGGACAGCTGAACGCTGATCTTCGCA
C. elegans tbb-2	CCGGAGTTACCACCTGTCTCCGATTCCCAGGACAGCTCAATGCCGATCTCCGCA
C. elegans ben-1	CCGGAGTCACCACGTGCCTCCGCTTCCCAGGACAACTCAATGCTGATCTCCGCA
H. contortus isotype1	CTGGTGTCACGACCTGCCTTCGATTCCCTGGACAGCTGAATGCTGATCTTCGCA
H. contortus isotype2	CTGGAGTAACAACATGCTTGCGATTCCCTGGACAACTAAATGCTGATCTACGTA
W. bancrofti	CTGGAGTAACAACTTGCTTACGTTTTCCTGGACAGTTG
T. trichiura	CTGGAGTAACGACATGCCTACGCTTTCCTGGTCAGTTGAATGCTGATTTGCGGA
T. circumcincta	CTGGAGTCACGACCTGCCTTCGATTCCCTGGACAGCTGAATGCTGATCTTCGCA
O. volvulus	CTGGAGTGACAACATGCTTGCGTTTTCCTGGACAGTTAAATGCCGATCTTCGTA
N. americanus	CTGGTGTTACTACCTGTCTTCGCTTCCCCGGTCAGTTGAATGCTGATCTTCGTA
B. malayi	CGGGAGTAACAACTTGCTTACGTTTCCCTGGACAGTTGAACGCCGATCTCCGTA
A. lumbricoides	CTGGCGTCACAACGTGCCTGCGCTTCCCTGGCCAGCTGAATGCAGACTTGCGCA
A. duodenale	CCGGTGTCACTACCTGCCTTCGCTTCCCTGGCCAGTTGAATGCTGATCTCCGCA

	820 – 860
C. oncophora isotype1	AGTTGGCCGTGAACATGGTTCCATTCCCTCGTCTTCACTTCTTTATGCCCGGTT
C. oncophora isotype2	AACTGGCTGTCAACATGGTGCCGTTCCCTCGCTTGCATTTCTTCATGCCTGGAT
C. elegans tbb-1	AGTTGGCTGTCAACATGGTTCCATTCCCACGTCTTCATTTCTTCATGCCTGGAT
C. elegans tbb-2	AGCTTGCCGTCAACATGGTTCCATTCCCACGTCTTCACTTCTTCATGCCAGGAT
C. elegans ben-1	AACTTGCAGTCAACATGGTTCCATTCCCACGTCTTCACTTCTTTATGCCAGGAT
H. contortus isotype1	AGTTAGCCGTGAACATGGTTCCATTCCCTCGTCTTCACTTCTTCATGCCCGGTT
H. contortus isotype2	AACTGGCTGTCAACATGGTGCCCTTCCCTCGGCTACATTTCTTCATGCCTGGAT
W. bancrofti	
T. trichiura	AGCTGGCAGTCAATATGGTGCCTTTTCCACGTTTGCATTTCTTTATGCCTGGCT
T. circumcincta	AGTTAGCCGTGAACATGGTTCCATTCCCTCGTCTTCACTTCTTCATGCCAGGTT
O. volvulus	AGCTCGCTGTTAATATGGTACCATTCCCACGATTGCATTTCTTCATGCCAGGAT
N. americanus	AGTTGGCTGTCAACATGGTTCCATTCCCACGTCTTCACTTCTTCATGCCTGGTT
B. malayi	AACTTGCCGTCAATATGGTACCATTTCCACGGTTGCATTTCTTTATGCCAGGAT
A. lumbricoides	AATTGGCAGTCAATATGGTGCCCTTCCCACGTCTTCACTTCTTCATGCCAGGTT
A. duodenale	AATTGGCTGTAAACATGGTTCCATTCCCACGTCTTCACTTCTTCATGCCTGGAT

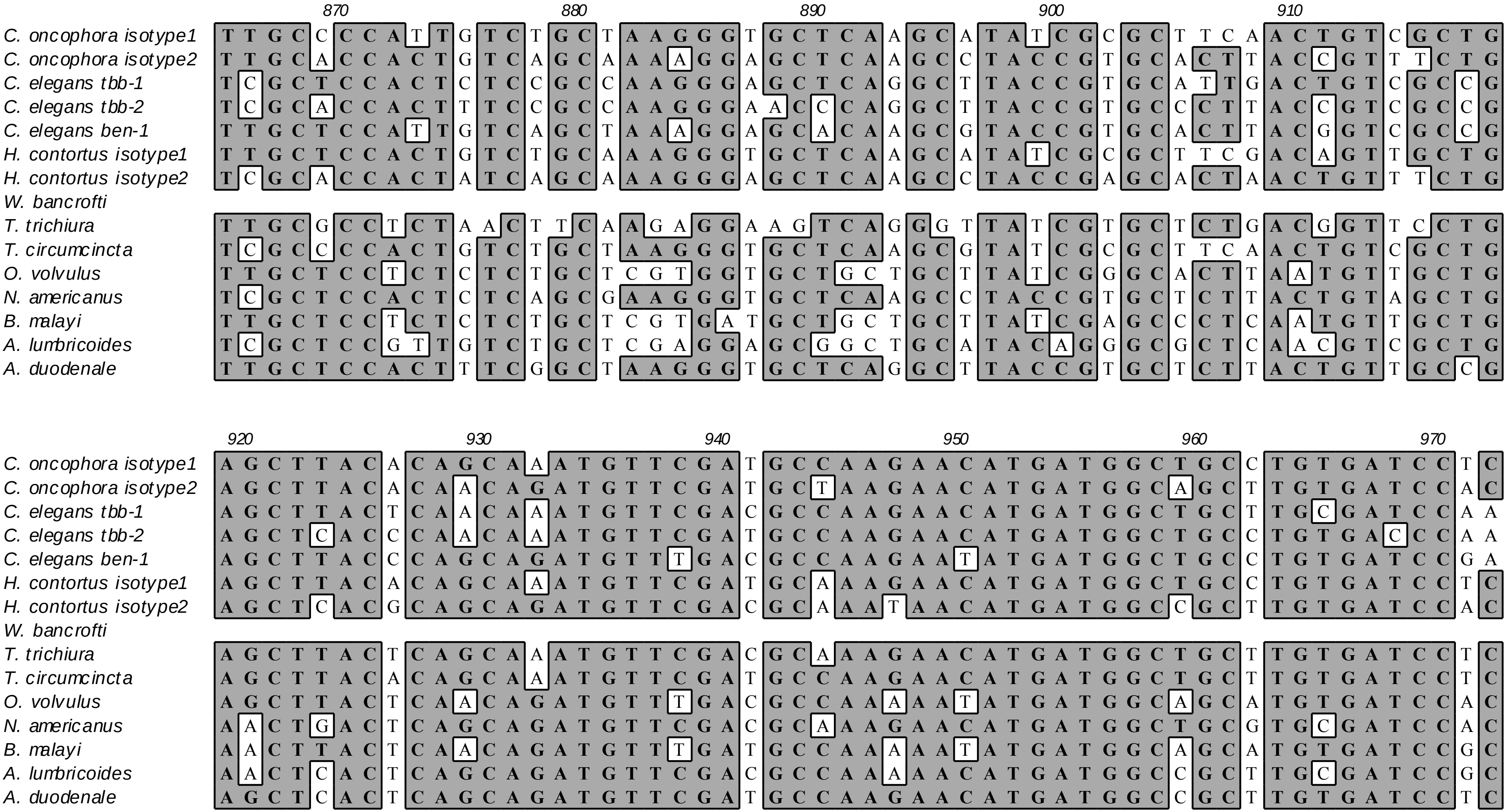
870 880 890 900 910
C. oncophora isotype1 TTGCCCCATTGTCTGCTAAGGGTGCTCAAGCATATCGCGCTTCAACTGTCGCTG
C. oncophora isotype2 TTGCACCACTGTCAGCAAAAGGAGCTCAAGCCTACCGTGCACTTACCGTTTCTG
C. elegans tbb-1 TCGCTCCACTCTCCGCCAAGGGAGCTCAGGCTTACCGTGCATTGACTGTCGCCG
C. elegans tbb-2 TCGCACCACTTTCCGCCAAGGGAACCCAGGCTTACCGTGCCCTTACCGTCGCCG
C. elegans ben-1 TTGCTCCATTGTCAGCTAAAGGAGCACAAGCGTACCGTGCACTTACGGTCGCCG
H. contortus isotype1 TTGCTCCACTGTCTGCAAAGGGTGCTCAAGCATATCGCGCTTCGACAGTTGCTG
H. contortus isotype2 TCGCACCACTATCAGCAAAGGGAGCTCAAGCCTACCGAGCACTAACTGTTTCTG
W. bancrofti
T. trichiura TTGCGCCTCTAACTTCAAGAGGAAGTCAGGGTTATCGTGCTCTGACGGTTCCTG
T. circumcincta TCGCCCCACTGTCTGCTAAGGGTGCTCAAGCGTATCGCGCTTCAACTGTCGCTG
O. volvulus TTGCTCCTCTCTCTGCTCGTGGTGCTGCTGCTTATCGGGCACTTAATGTTGCTG
N. americanus TCGCTCCACTCTCAGCGAAGGGTGCTCAAGCCTACCGTGCTCTTACTGTAGCTG
B. malayi TTGCTCCTCTCTCTGCTCGTGATGCTGCTGCTTATCGAGCCCTCAATGTTGCTG
A. lumbricoides TCGCTCCGTTGTCTGCTCGAGGAGCGGCTGCATACAGGGCGCTCAACGTCGCTG
A. duodenale TTGCTCCACTTTCGGCTAAGGGTGCTCAGGCTTACCGTGCTCTTACTGTTGCCG
920 930 940 950 960 970
C. oncophora isotype1 AGCTTACACAGCAAATGTTCGATGCCAAGAACATGATGGCTGCCTGTGATCCTC
C. oncophora isotype2 AGCTTACACAACAGATGTTCGATGCTAAGAACATGATGGCAGCTTGTGATCCAC
C. elegans tbb-1 AGCTTACTCAACAAATGTTCGACGCCAAGAACATGATGGCTGCTTGCGATCCAA
C. elegans tbb-2 AGCTCACCCAACAAATGTTCGATGCCAAGAACATGATGGCTGCCTGTGACCCAA
C. elegans ben-1 AGCTTACCCAGCAGATGTTTGACGCCAAGAATATGATGGCTGCCTGTGATCCGA
H. contortus isotype1 AGCTTACACAGCAAATGTTCGATGCAAAGAACATGATGGCTGCCTGTGATCCTC
H. contortus isotype2 AGCTCACGCAGCAGATGTTCGACGCAAATAACATGATGGCCGCTTGTGATCCAC
W. bancrofti
T. trichiura AGCTTACTCAGCAAATGTTCGACGCAAAGAACATGATGGCTGCTTGTGATCCTC
T. circumcincta AGCTTACACAGCAAATGTTCGATGCCAAGAACATGATGGCTGCTTGTGATCCTC
O. volvulus AGCTTACTCAACAGATGTTTGACGCCAAAAATATGATGGCAGCATGTGATCCAC
N. americanus AACTGACTCAGCAGATGTTCGACGCAAAGAACATGATGGCTGCGTGCGATCCAC
B. malayi AACTTACTCAACAGATGTTTGATGCCAAAAATATGATGGCAGCATGTGATCCGC
A. lumbricoides AACTCACTCAGCAGATGTTCGACGCCAAAAACATGATGGCCGCTTGCGATCCGC
A. duodenale AGCTCACTCAGCAGATGTTCGATGCCAAGAACATGATGGCCGCTTGTGATCCTC

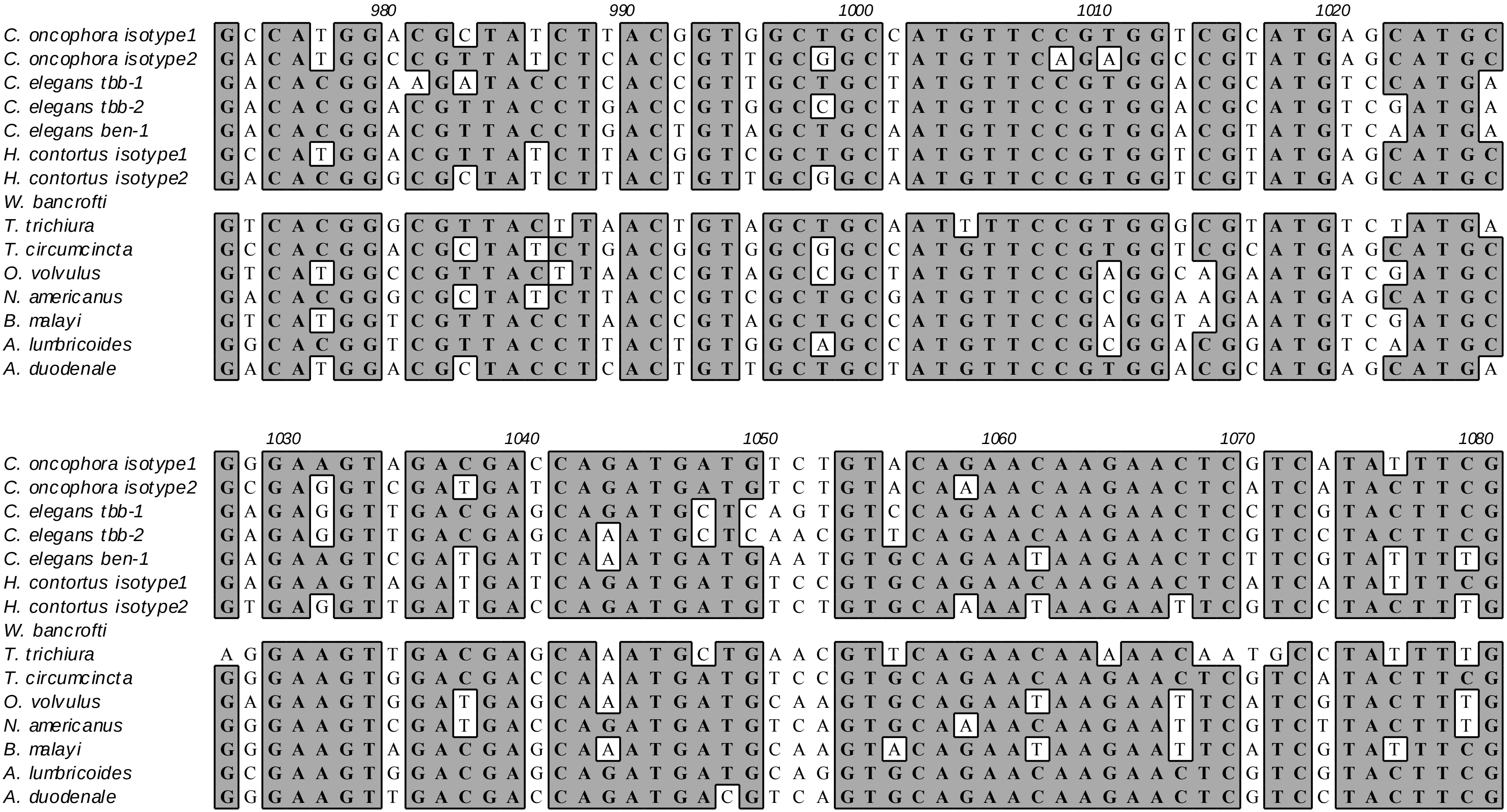
980
990
1000
1010
1020
C. oncophora isotype1 GCCATGGACGCTATCTTACGGTGGCTGCCATGTTCCGTGGTCGCATGAGCATGC
C. oncophora isotype2 GACATGGCCGTTATCTCACCGTTGCGGCTATGTTCAGAGGCCGTATGAGCATGC
C. elegans tbb-1 GACACGGAAGATACCTCACCGTTGCTGCTATGTTCCGTGGACGCATGTCCATGA
C. elegans tbb-2 GACACGGACGTTACCTGACCGTGGCCGCTATGTTCCGTGGACGCATGTCGATGA
C. elegans ben-1 GACACGGACGTTACCTGACTGTAGCTGCAATGTTCCGTGGACGTATGTCAATGA
H. contortus isotype1 GCCATGGACGTTATCTTACGGTCGCTGCTATGTTCCGTGGTCGTATGAGCATGC
H. contortus isotype2 GACACGGGCGCTATCTTACTGTTGCGGCAATGTTCCGTGGTCGTATGAGCATGC
W. bancrofti
T. trichiura GTCACGGGCGTTACTTAACTGTAGCTGCAATTTTCCGTGGGCGTATGTCTATGA
T. circumcincta GCCACGGACGCTATCTGACGGTGGCGGCCATGTTCCGTGGTCGCATGAGCATGC
O. volvulus GTCATGGCCGTTACTTAACCGTAGCCGCTATGTTCCGAGGCAGAATGTCGATGC
N. americanus GACACGGGCGCTATCTTACCGTCGCTGCGATGTTCCGCGGAAGAATGAGCATGC
B. malayi GTCATGGTCGTTACCTAACCGTAGCTGCCATGTTCCGAGGTAGAATGTCGATGC
A. lumbricoides GGCACGGTCGTTACCTTACTGTGGCAGCCATGTTCCGCGGACGGATGTCAATGC
A. duodenale GACATGGACGCTACCTCACTGTTGCTGCTATGTTCCGTGGACGCATGAGCATGA
1030
1040
1050
1060
1070
1080
C. oncophora isotype1 GGGAAGTAGACGACCAGATGATGTCTGTACAGAACAAGAACTCGTCATATTTCG
C. oncophora isotype2 GCGAGGTCGATGATCAGATGATGTCTGTACAAAACAAGAACTCATCATACTTCG
C. elegans tbb-1 GAGAGGTTGACGAGCAGATGCTCAGTGTCCAGAACAAGAACTCCTCGTACTTCG
C. elegans tbb-2 GAGAGGTTGACGAGCAAATGCTCAACGTTCAGAACAAGAACTCGTCCTACTTCG
C. elegans ben-1 GAGAAGTCGATGATCAAATGATGAATGTGCAGAATAAGAACTCTTCGTATTTTG
H. contortus isotype1 GAGAAGTAGATGATCAGATGATGTCCGTGCAGAACAAGAACTCATCATATTTCG
H. contortus isotype2 GTGAGGTTGATGACCAGATGATGTCTGTGCAAAATAAGAATTCGTCCTACTTTG
W. bancrofti
T. trichiura AGGAAGTTGACGAGCAAATGCTGAACGTTCAGAACAAAAACAATGCCTATTTTG
T. circumcincta GGGAAGTGGACGACCAAATGATGTCCGTGCAGAACAAGAACTCGTCATACTTCG
O. volvulus GAGAAGTGGATGAGCAAATGATGCAAGTGCAGAATAAGAATTCATCGTACTTTG
N. americanus GGGAAGTCGATGACCAGATGATGTCAGTGCAAAACAAGAATTCGTCTTACTTTG
B. malayi GGGAAGTAGACGAGCAAATGATGCAAGTACAGAATAAGAATTCATCGTATTTCG
A. lumbricoides GCGAAGTGGACGAGCAGATGATGCAGGTGCAGAACAAGAACTCGTCGTACTTCG
A. duodenale GGGAAGTTGACGACCAGATGACGTCAGTGCAGAACAAGAACTCGTCCTACTTCG

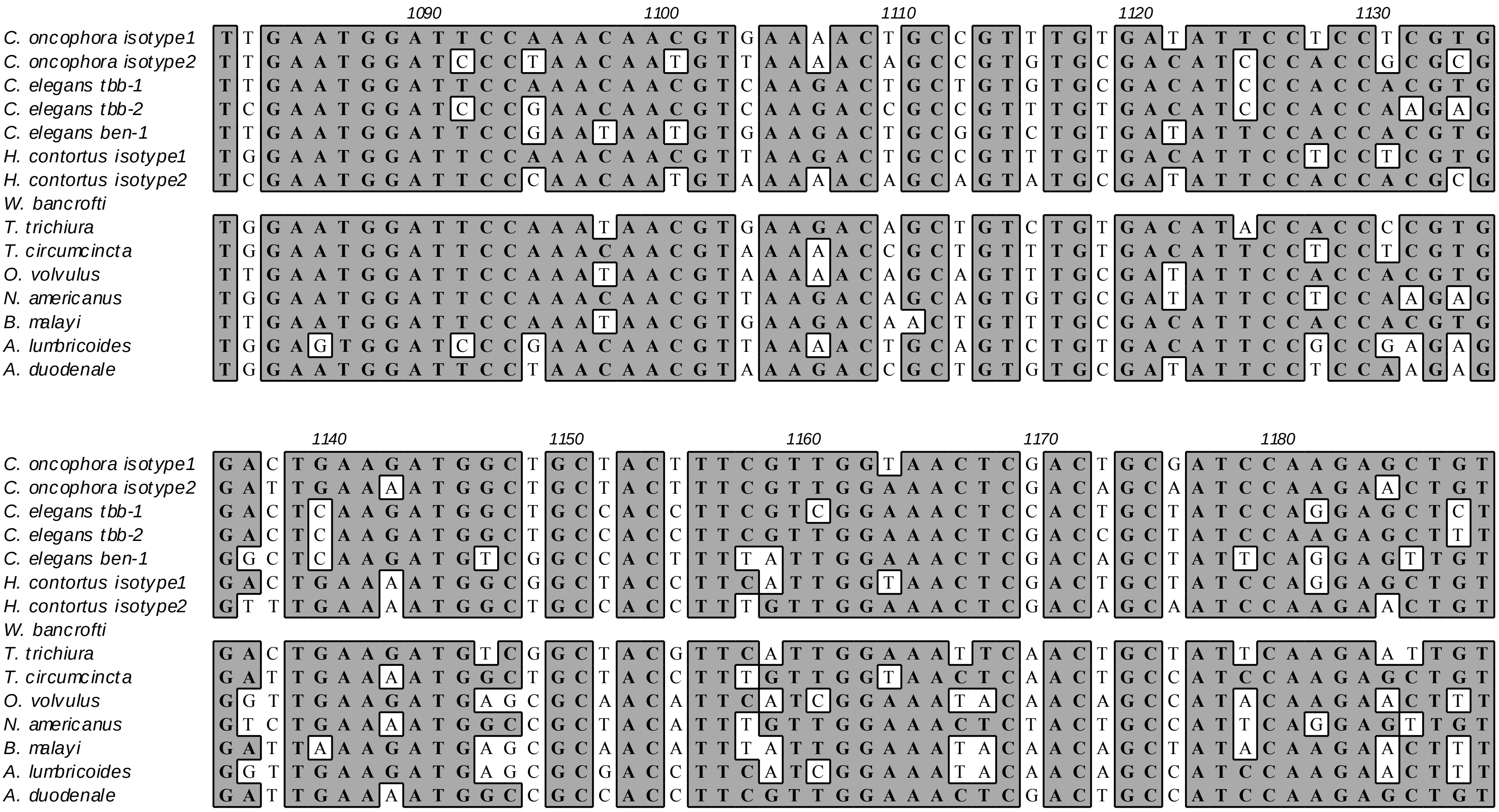
1090
1100
1110
1120
1130
C. oncophora isotype1
C. oncophora isotype2
C. elegans tbb-1
C. elegans tbb-2
C. elegans ben-1
H. contortus isotype1
H. contortus isotype2
W. bancrofti
T. trichiura
T. circumcincta
O. volvulus
N. americanus
B. malayi
A. lumbricoides
A. duodenale
1140
1150
1160
1170
1180
C. oncophora isotype1
C. oncophora isotype2
C. elegans tbb-1
C. elegans tbb-2
C. elegans ben-1
H. contortus isotype1
H. contortus isotype2
W. bancrofti
T. trichiura
T. circumcincta
O. volvulus
N. americanus
B. malayi
A. lumbricoides
A. duodenale

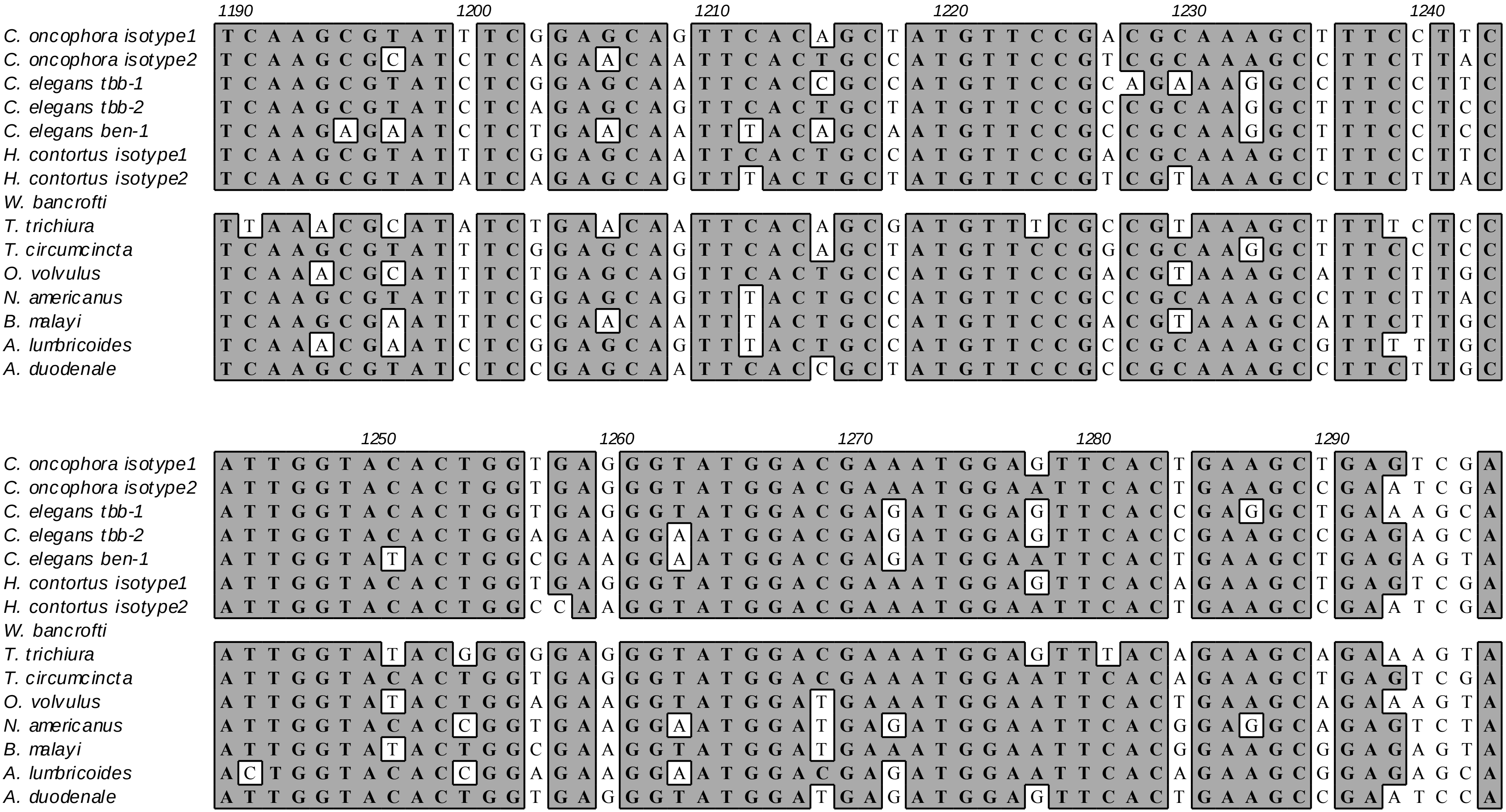
1190 1200 1210 1220 1230 1240
C. oncophora isotype1 TCAAGCGTATTTCGGAGCAGTTCACAGCTATGTTCCGACGCAAAGCTTTCCTTC
C. oncophora isotype2 TCAAGCGCATCTCAGAACAATTCACTGCCATGTTCCGTCGCAAAGCCTTCTTAC
C. elegans tbb-1 TCAAGCGTATCTCGGAGCAATTCACCGCCATGTTCCGCAGAAAGGCCTTCCTTC
C. elegans tbb-2 TCAAGCGTATCTCAGAGCAGTTCACTGCTATGTTCCGCCGCAAGGCTTTCCTCC
C. elegans ben-1 TCAAGAGAATCTCTGAACAATTTACAGCAATGTTCCGCCGCAAGGCTTTCCTCC
H. contortus isotype1 TCAAGCGTATTTCGGAGCAATTCACTGCCATGTTCCGACGCAAAGCTTTCCTTC
H. contortus isotype2 TCAAGCGTATATCAGAGCAGTTTACTGCTATGTTCCGTCGTAAAGCCTTCTTAC
W. bancrofti
T. trichiura TTAAACGCATATCTGAACAATTCACAGCGATGTTTCGCCGTAAAGCTTTTCTCC
T. circumcincta TCAAGCGTATTTCGGAGCAGTTCACAGCTATGTTCCGGCGCAAGGCTTTCCTCC
O. volvulus TCAAACGCATTTCTGAGCAGTTCACTGCCATGTTCCGACGTAAAGCATTCTTGC
N. americanus TCAAGCGTATTTCGGAGCAGTTTACTGCCATGTTCCGCCGCAAAGCCTTCTTAC
B. malayi TCAAGCGAATTTCCGAACAATTTACTGCCATGTTCCGACGTAAAGCATTCTTGC
A. lumbricoides TCAAACGAATCTCGGAGCAGTTTACTGCCATGTTCCGCCGCAAAGCGTTTTTGC
A. duodenale TCAAGCGTATCTCCGAGCAATTCACCGCTATGTTCCGCCGCAAAGCCTTCTTGC
1250 1260 1270 1280 1290
C. oncophora isotype1 ATTGGTACACTGGTGAGGGTATGGACGAAATGGAGTTCACTGAAGCTGAGTCGA
C. oncophora isotype2 ATTGGTACACTGGTGAGGGTATGGACGAAATGGAATTCACTGAAGCCGAATCGA
C. elegans tbb-1 ATTGGTACACTGGTGAGGGTATGGACGAGATGGAGTTCACCGAGGCTGAAAGCA
C. elegans tbb-2 ATTGGTACACTGGAGAAGGAATGGACGAGATGGAGTTCACCGAAGCCGAGAGCA
C. elegans ben-1 ATTGGTATACTGGCGAAGGAATGGACGAGATGGAATTCACTGAAGCTGAGAGTA
H. contortus isotype1 ATTGGTACACTGGTGAGGGTATGGACGAAATGGAGTTCACAGAAGCTGAGTCGA
H. contortus isotype2 ATTGGTACACTGGCCAAGGTATGGACGAAATGGAATTCACTGAAGCCGAATCGA
W. bancrofti
T. trichiura ATTGGTATACGGGGGAGGGTATGGACGAAATGGAGTTTACAGAAGCAGAAAGTA
T. circumcincta ATTGGTACACTGGTGAGGGTATGGACGAAATGGAATTCACAGAAGCTGAGTCGA
O. volvulus ATTGGTATACTGGAGAAGGTATGGATGAAATGGAATTCACTGAAGCAGAAAGTA
N. americanus ATTGGTACACCGGTGAAGGAATGGATGAGATGGAATTCACGGAGGCAGAGTCTA
B. malayi ATTGGTATACTGGCGAAGGTATGGATGAAATGGAATTCACGGAAGCGGAGAGTA
A. lumbricoides ACTGGTACACCGGAGAAGGAATGGACGAGATGGAATTCACAGAAGCGGAGAGCA
A. duodenale ATTGGTACACTGGTGAGGGTATGGATGAGATGGAGTTCACTGAAGCCGAATCCA

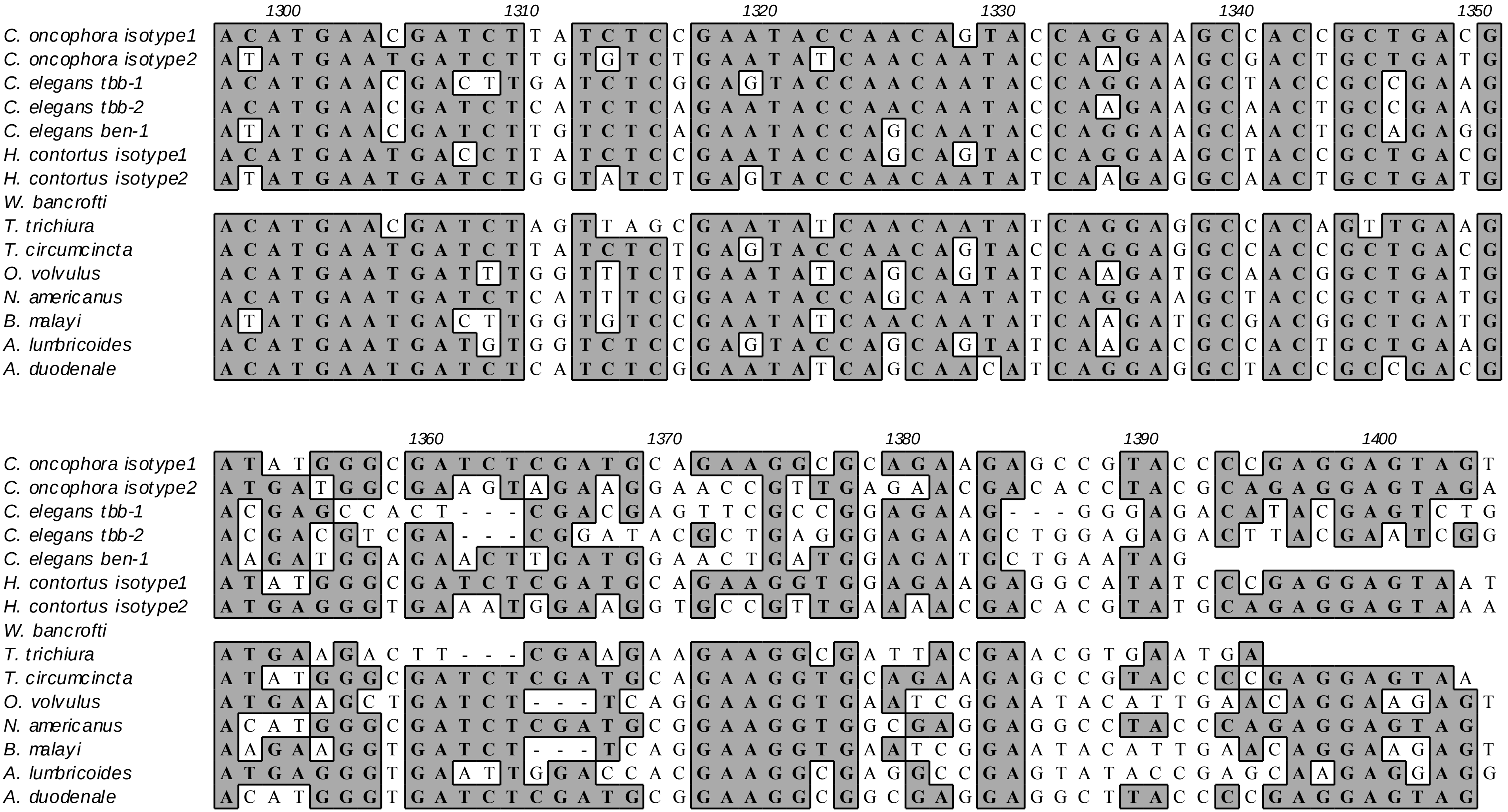
1300 1310 1320 1330 1340 1350
C. oncophora isotype1 ACATGAACGATCTTATCTCCGAATACCAACAGTACCAGGAAGCCACCGCTGACG
C. oncophora isotype2 ATATGAATGATCTTGTGTCTGAATATCAACAATACCAAGAAGCGACTGCTGATG
C. elegans tbb-1 ACATGAACGACTTGATCTCGGAGTACCAACAATACCAGGAAGCTACCGCCGAAG
C. elegans tbb-2 ACATGAACGATCTCATCTCAGAATACCAACAATACCAAGAAGCAACTGCCGAAG
C. elegans ben-1 ATATGAACGATCTTGTCTCAGAATACCAGCAATACCAGGAAGCAACTGCAGAGG
H. contortus isotype1 ACATGAATGACCTTATCTCCGAATACCAGCAGTACCAGGAAGCTACCGCTGACG
H. contortus isotype2 ATATGAATGATCTGGTATCTGAGTACCAACAATATCAAGAGGCAACTGCTGATG
W. bancrofti
T. trichiura ACATGAACGATCTAGTTAGCGAATATCAACAATATCAGGAGGCCACAGTTGAAG
T. circumcincta ACATGAATGATCTTATCTCTGAGTACCAACAGTACCAGGAGGCCACCGCTGACG
O. volvulus ACATGAATGATTTGGTTTCTGAATATCAGCAGTATCAAGATGCAACGGCTGATG
N. americanus ACATGAATGATCTCATTTCGGAATACCAGCAATATCAGGAAGCTACCGCTGATG
B. malayi ATATGAATGACTTGGTGTCCGAATATCAACAATATCAAGATGCGACGGCTGATG
A. lumbricoides ACATGAATGATGTGGTCTCCGAGTACCAGCAGTATCAAGACGCCACTGCTGAAG
A. duodenale ACATGAATGATCTCATCTCGGAATATCAGCAACATCAGGAGGCTACCGCCGACG
1360 1370 1380 1390 1400
C. oncophora isotype1 ATATGGGCGATCTCGATGCAGAAGGCGCAGAAGAGCCGTACCCCGAGGAGTAGT
C. oncophora isotype2 ATGATGGCGAAGTAGAAGGAACCGTTGAGAACGACACCTACGCAGAGGAGTAGA
C. elegans tbb-1 ACGAGCCACT---CGACGAGTTCGCCGGAGAAG---GGGAGACATACGAGTCTG
C. elegans tbb-2 ACGACGTCGA---CGGATACGCTGAGGGAGAAGCTGGAGAGACTTACGAATCGG
C. elegans ben-1 AAGATGGAGAACTTGATGGAACTGATGGAGATGCTGAATAG
H. contortus isotype1 ATATGGGCGATCTCGATGCAGAAGGTGGAGAAGAGGCATATCCCGAGGAGTAAT
H. contortus isotype2 ATGAGGGTGAAATGGAAGGTGCCGTTGAAACGACACGTATGCAGAGGAGTAAA
W. bancrofti
T. trichiura ATGAAGACTT---CGAAGAAGAAGGCGATTACGAACGTGAATGA
T. circumcincta ATATGGGCGATCTCGATGCAGAAGGTGCAGAAGAGCCGTACCCCGAGGAGTAA
O. volvulus ATGAAGCTGATCT---TCAGGAAGGTGAATCGGAATACATTGAACAGGAAGAGT
N. americanus ACATGGGCGATCTCGATGCGGAAGGTGGCGAGGAGGCCTACCCAGAGGAGTAG
B. malayi AAGAAGGTGATCT---TCAGGAAGGTGAATCGGAATACATTGAACAGGAAGAGT
A. lumbricoides ATGAGGGTGAATTGGACCACGAAGGCGAGGCCGAGTATACCGAGCAAGAGGAGG
A. duodenale ACATGGGTGATCTCGATGCGGAAGGCGGCGAGGAGGCTTACCCCGAGGAGTAG

	1410 · 1420 · 1430 · 1440 · 1450
C. oncophora isotype1	G - A T C T A C C C G T G T T G C G C T G T - - - T T T T T C T G T G T C A A T G C G A A A T A C A C A T T
C. oncophora isotype2	A - A A G C A A T T G C C C A A T T C A C T - A T G C A T C T C A T T T T T T T G C A G C T A T C A T A C T
C. elegans tbb-1	A G C A A T A A T C T G T G T A T C A A T C A C T A A A T T A T C C T G C C C C G T T T C G T A A T T T C C
C. elegans tbb-2	A G C A A T A A A T G C A A G A T C C T T T C A A G C A T T C C C T T C T T C T C T A T C A C T C T T C T T
C. elegans ben-1	
H. contortus isotype1	G - A T C C A C A A A - G T T G T G C T C T - - - T T T T C C T G T G T C A A T G C G A A A T A C A C A T T
H. contortus isotype2	G G A A G C A T C T G C C C A A C T C A T T T A T G C A T C T C A T T C T T - - G C G G C T A T T G T A T T
W. bancrofti	
T. trichiura	
T. circumcincta	
O. volvulus	A A
N. americanus	
B. malayi	G A G C A C A A A G T G T C A G C T T T G T G A A A A A A G A A C T T T T A C A G T A T T T T A T T T A T C
A. lumbricoides	A G T G A
A. duodenale	

	1460 · 1470 · 1480 · 1490 · 1500 · 1510
C. oncophora isotype1	G G T T G C G T T
C. oncophora isotype2	G C A T A C G T T G T A A A A G T T T C T C T G A A C G G C T G T T C T C A A A G C A C T T T C T A T T T G
C. elegans tbb-1	T G T A C A - A T G A T C T T T T C T T T C G C C C A C T C C C A A T A T T T T T A T G A C T C G A A T G A
C. elegans tbb-2	T C T T T T T G T C A A A A A A T T C T C T C G C T A A T T T A T T T G C T T T T T T A A T G T T A T T A T
C. elegans ben-1	
H. contortus isotype1	G G T T G C G T T G T G T A T G G T G T T A T T A A A G C T T G C T A A T T G A T A A A G T T A A T G A A G
H. contortus isotype2	G C G C G T A C T G
W. bancrofti	
T. trichiura	
T. circumcincta	
O. volvulus	
N. americanus	
B. malayi	G T C T T T T C C A T G T T T T T A T G T T G T T A C A T T T T A T T T T G T T A C T C G C T T T C G A C A
A. lumbricoides	
A. duodenale	

	1520 · 1530 · 1540 · 1550 · 1560
C. oncophora isotype1	
C. oncophora isotype2	T G A T T T T A T T A T G A T C T A T T C T T G C T G C T A A T T A T T T C T T C G C G A G C T T C C C G A
C. elegans tbb-1	A C G A T G A T T A A T T T
C. elegans tbb-2	T T T A T G A C T T T T T A T A G T C A C T G A A A A G T T T G C A T C T G A G T G A A G T G A A T G C T A
C. elegans ben-1	
H. contortus isotype1	T T T A A A A A A A A A A A A A A A G A A
H. contortus isotype2	
W. bancrofti	
T. trichiura	
T. circumcincta	
O. volvulus	
N. americanus	
B. malayi	T A T A T T A G C A A A T T T T A A A G A A T T A T A G C G A C A A A T C T C A T T G T T G T A C T T C T C
A. lumbricoides	
A. duodenale	

	1570 · 1580 · 1590 · 1600 · 1610 · 1620
C. oncophora isotype1	
C. oncophora isotype2	T G A G T T T G A T A A A G C G A A T G A T C A G G C T G A
C. elegans tbb-1	
C. elegans tbb-2	T C A A A A T G T G A T T C T G T C T G A T G T A C T T T C A C A A T C T C T C T T C A A T T C C A T T T T
C. elegans ben-1	
H. contortus isotype1	
H. contortus isotype2	
W. bancrofti	
T. trichiura	
T. circumcincta	
O. volvulus	
N. americanus	
B. malayi	A A T A A A T T A C
A. lumbricoides	
A. duodenale	

	1630 1640 1650 1660 1670
C. oncophora isotype1	
C. oncophora isotype2	
C. elegans tbb-1	
C. elegans tbb-2	G A A G T G C T T T A A A C C C G A A A G G T T G A G A A A A A T G C G A G C G C T C A A A T A T T T G T A
C. elegans ben-1	
H. contortus isotype1	
H. contortus isotype2	
W. bancrofti	
T. trichiura	
T. circumcincta	
O. volvulus	
N. americanus	
B. malayi	
A. lumbricoides	
A. duodenale	

	1680 1690 1700 1710 1720
C. oncophora isotype1	
C. oncophora isotype2	
C. elegans tbb-1	
C. elegans tbb-2	T T G T G T T C G T T G A G T G A C C C A A C A A A A A G A G G A A A
C. elegans ben-1	
H. contortus isotype1	
H. contortus isotype2	
W. bancrofti	
T. trichiura	
T. circumcincta	
O. volvulus	
N. americanus	
B. malayi	
A. lumbricoides	
A. duodenale	

The alignment shows a high conservation in all beta-tubulin cDNA sequences. The square shows codon 200 where “TTC” is present in all sequences except for *C. elegans* tbb-1 and tbb-2
